# Parental domestic violence and abuse, mental ill-health, and substance misuse and the impact on child mental health: a secondary data analysis using the UK Millennium Cohort Study

**DOI:** 10.1186/s12889-024-19694-1

**Published:** 2024-08-26

**Authors:** Kate Allen, G. J. Melendez-Torres, Tamsin Ford, Chris Bonell, Vashti Berry

**Affiliations:** 1 University of Exeter, South Cloisters, St Luke’s Campus, College Road, Exeter, EX1 1TE UK; 2 Department of Psychiatry, University of Cambridge, Herchel Smith Building, Forvie Site, Robinson Way, Cambridge, CB2 0SZ UK; 3 London School of Hygiene and Tropical Medicine, 15-17 Tavistock Place, London, WC1H 9SH UK

**Keywords:** Adverse childhood experiences, Domestic violence and abuse, Mental ill-health, Substance misuse, Child mental health, Latent variable mixture modelling

## Abstract

**Background:**

Parental domestic violence and abuse (DVA), mental ill-health (MH), and substance misuse (SU) can have a negative impact on both parents and children. However, it remains unclear if and how parental DVA, MH, and SU cluster and the impacts this clustering might have. We examined *how* parental DVA, MH, and SU cluster during early childhood, the demographic/contextual profiles of these clusters, and how these clusters relate to child MH trajectories.

**Methods:**

We examined data from 15,377 families in the UK Millennium Cohort Study. We used: (1) latent class analysis to create groups differentially exposed to parental DVA, MH, and SU at age three; (2) latent growth curve modelling to create latent trajectories of child MH from ages 3–17; and (3) a case-weight approach to relate latent classes to child MH trajectories.

**Results:**

We identified three latent classes: *high-frequency alcohol use* (11.9%), *elevated adversity* (3.5%), and *low-level adversity* (84.6%). Children in the *elevated adversity* class had higher probabilities of being from low-socioeconomic backgrounds and having White, younger parents. Children exposed to *elevated adversity* displayed worse MH at age three (intercept = 2.274; *p* < 0.001) compared the *low-level adversity* (intercept = 2.228; *p* < 0.001) and *high-frequency alcohol use* class (intercept = 2.068; *p* < 0.001). However, latent growth factors (linear and quadratic terms) of child MH did not differ by latent class.

**Conclusions:**

Parental DVA, MH, and SU cluster during early childhood and this has a negative impact on children’s MH at age three, leading to similar levels of poor MH across time. Intervening early to prevent the initial deterioration, using a syndemic-approach is essential.

**Supplementary Information:**

The online version contains supplementary material available at 10.1186/s12889-024-19694-1.

## Background

Parental domestic violence and abuse (DVA), mental ill-health (MH), and substance misuse (SU; including alcohol and drug use) are prevalent public health problems both in the UK [1–3] and worldwide [4–7] and are considered to be three of several adverse childhood experiences (ACEs). When considered in combination, an estimated 3.6% of UK children are living in households where all three of parental DVA, MH, and SU are present [8]. This is a conservative estimate, with epidemiological studies, qualitative accounts, and referrals to specialist helplines indicating that each of these three ACEs have been exacerbated by COVID-19 government-related restrictions [9]. Unsurprisingly, parental DVA, MH, and SU can have a negative impact on parenting capacity, child internalising and externalising behaviour, and a range of child health outcomes across the life course [10], with each of parental DVA, MH, and SU being associated with increased risk of child maltreatment [11, 12]. Furthermore, evidence suggests these ACEs tend to be intergenerational, with those who experience parental DVA, MH, and SU during childhood being at increased risk of developing problems with violence, MH, and SU themselves, later on in life [13].

Families experiencing these three ACEs are likely to be at increased risk of harm, with evidence suggesting that each of these public health problems are likely to modify the risk of the other occurring [14–16]. Consequently, improving the ways in which we prevent and respond to parental DVA, MH, and SU is a policy and practice priority within the UK [17–19]. Although families may experience these issues in combination, commissioning and service provision for DVA, MH, and SU have remained historically siloed, resulting in difficulties providing integrated support [20]. Moreover, we lack effective evidence-based interventions for these difficulties in combination [21]. Developing an effective response requires an understanding of *how* these ACEs cluster, the demographic/contextual profiles of these clusters, and whether clustering impacts child outcomes.

Few studies have considered this, with research to date focusing on each of these ACEs in isolation or cumulatively alongside other ACEs which ignores potential synergistic relationships and effects interventions may need to target [22, 23]. Where recent studies have considered *how* ACEs cluster, findings remained limited or mixed. For example, when considering a whole range of ACEs (e.g., child maltreatment, parental DVA, parental MH, parental SU, parental separation/divorce, parental convictions, death of close family members etc.) there is mixed evidence to suggest parental DVA, MH, and SU form a prominent cluster (e.g. [24–26], When focusing on parental DVA, MH, and SU specifically, a recent systematic review identified only three studies [27–29] that had explored the relationship between these three ACEs and the impact they might have on child abuse and behavioural outcomes [30]. This research has also tended to focus on high-risk samples, retrospective reports, or maternal experiences of DVA, MH, and SU rather than experiences within the *whole family* context. Taking a family systems perspective, these ACEs are likely to impact children regardless of who is experiencing them.

To address this gap, Adjei et al. [31] considered how parental DVA, MH, alcohol use, and poverty cluster across 9-months to 14-years of age and how these clustered-trajectories might relate to child outcomes at age 14. Using prospective data from the UK Millennium Cohort Study (MCS), they identified six clustered-trajectories including: (1) *“no adversities”*, (2) *“persistent poverty”*, (3) *“persistent MH”*, (4) *“persistent alcohol use”*, (5) *“persistent DVA”*, (6) *“persistent poverty and MH”*. Group membership in the *“persistent poverty and MH”* trajectory was related to the most negative socioemotional, cognitive disability, drug experimentation, and obesity outcomes at age 14, suggesting important targets for future intervention. Although providing a valuable insight into how ACE trajectories co-occur, it tells us little about how adversity clusters during potentially sensitive time-periods in a child’s development. While the evidence base is still developing, there is some evidence to suggest that experiencing ACEs during early childhood (as opposed to late childhood or adolescence) is more likely to have negative impacts on a child across their life course when considering specific outcomes [32, 33]. Furthermore, policy and practice often prioritise this period of child development as a crucial time for prevention and early intervention [34]. Exploring how parental DVA, MH, and SU cluster during such time points will help further advance our understanding of ACE clusters whilst informing our timing of intervention efforts [32]. To our knowledge, no studies have yet considered this.

Drawing on our recent systematic review [21] and literature from the individual fields of DVA, MH, and SU [35–39], important demographic and contextual characteristics that are likely to relate to the co-occurrence of parental DVA, MH, and SU include socio-demographic characteristics such as parental age, ethnicity, education/qualifications, household income, number of children, and housing tenure. However, our understanding of how these characteristics might relate to specific patterning of parental DVA, MH, and SU remains limited. Exploring the demographic and contextual profiles of families experiencing specific clusters of parental DVA, MH, and SU during early childhood is essential to help inform the development of preventive interventions (both in terms of who preventive interventions for clustered parental DVA, MH, and SU might seek to target and how preventive interventions might need to be tailored), as well as inform policymakers. Furthermore, it could help elucidate avenues for future research, helping us to develop a more nuanced understanding of the co-occurrence of parental DVA, MH, and SU.

We examined *how* parental DVA, MH, and SU cluster during early childhood, the demographic/contextual profiles of these clusters, and how these clusters might relate to child trajectories of MH over time. We focus on trajectories of child MH because child MH problems can lead to a range of negative outcomes later on in life including poor academic attainment, interpersonal difficulties, substance use, and physical health problems [40] and therefore, intervening early is essential. Overall, our work seeks to clarify *who* an intervention for parental DVA, MH, and SU should target, as well as *whether* clustered risk impacts children’s MH trajectories.

## Methods

### Study design

We conducted a latent variable mixture modelling analysis using data from the MCS; a freely accessible cohort study of ~ 19,000 UK children born between September 2000–January 2002 [41]. MCS cohort children were identified for recruitment via UK government child benefit records and eligible if they were living in the UK at 9-months of age. Oversampling of hard-to-reach groups resulted in a nationally representative sample [41]. MCS collected data on cohort children, and their families, in seven sweeps including when the child was; (1) 9-months-old; (2) 3-years-old; (3) 5-years-old; (4) 7-years-old; (5) 11-years-old; (6) 14-years-old; and (7) 17-years-old. Our study utilises exposure variables collected from main respondents (most often mothers/mother figures) and resident partner respondents (most often fathers/father figures) at age three and child outcome variables at ages 3–17. Most data were collected during home visits via face-to-face interview or self-completion [42–44]. Child outcome data at age 17 were also collected outside of home visits via self-completion [42]. We excluded multiple births from our study due to lack of independence. This left 15,377 cohort children and families who had provided at least some data at age three.

Our study was granted ethical approval from the University of Exeter College of Medicine and Health Ethics Committee (number: 489638). The protocol can be found on the Open Science Framework (https://osf.io/c6tbh/).

### Variables

#### Exposure: latent clusters of parental DVA, MH, alcohol use, and drug use

Latent clusters were created using binary manifest indicators representing the presence/absence of parental DVA, MH, alcohol use, and drug use at age three, derived using data from main and partner respondents (see Table 1 for overview and Supplementary Appendix S1, Additional file 1 for rationale). This was the earliest that data were available from main and partner respondents on all variables of interest.

**Table 1:** Overview of manifest indicators created using data collected from main and partner respondents at child age three

Manifest indicator	Item/scale description	Item/scale scoring	Manifest indicator scoring
Parental DVA (physical violence)	Main and partner respondents responded to a single question assessing the presence or absence of physical violence in their relationship: *“People often use force in a relationship – grabbing*,* pushing*,* shaking*,* hitting*, kicking etc. Has your *husband/wife/partner* ever used force on you for any reason?”	Yes, no, *do not want to answer*.	Yes (main **or** partner respondent = yes) vs. No (main **and** partner respondent = no).
Parental MH	Main and partner respondents completed the Kessler Psychological Distress Scale-6 (Kessler et al., 2010). The K6 is a six-item scale which measures non-specific psychological distress by assessing depressive and anxiety-related symptoms experienced in the last 30 days.	0–24 with higher scores indicating greater difficulties.	Yes (main **or** partner respondent = ≥ 13) vs. No (main **and** partner respondent = < 13).
Parental alcohol use (frequency)	Main and partner respondents responded to a single question assessing their frequency of alcohol use: *“Which of the following best describes how often you drink alcohol?”*	Every day, 5–6 times per week, 3–4 times per week, 1–2 times per week, 1–2 times per month, less than once a month, never, *refused*.	Yes (main **or** partner respondent = every day or 5–6 times a week) vs. No (main **and** partner respondent = 3–4 times per week, 1–2 times per week, 1–2 times per month, less than once a month, or never).
Parental drug use	Main and partner respondents responded to a single question assessing their drug use in the past year: *“During the past year have you used any recreational drugs like cannabis*,* cocaine or ecstasy?”*	Occasionally, regularly, never, *can’t say*.	Yes (main **or** partner respondent = occasionally or regularly) vs. No (main **and** partner respondent = never).

NB. Responses in italics including *‘do not want to answer’*, *‘refused’*, or *‘can’t say’* were recoded as ‘missing’

#### Outcome: latent trajectories of child MH

Our outcome variables were latent intercept and growth factors of parent-reported SDQ total difficulties (SDQ-TD) scores (Supplementary Appendix S1, Additional file 1) measured at ages 3–17. The SDQ is a 25-item measure of child behaviour which includes five subscales addressing emotional symptoms, conduct problems, hyperactivity/inattention, peer relationship problems and prosocial behaviour. Respondents respond to 25-items on a three-point Likert scale (‘not true’, ‘somewhat true’, or ‘certainly true’). Scores from the first four subscales are summed to create a ‘total difficulties’ score ranging from 0–40, with higher scores indicating greater difficulties [45]. The SDQ was completed by the main respondent from ages 3–14, and main or partner respondents at age 17 [42–44].

#### Demographic and contextual variables

Demographic and contextual variables included: child sex measured at age 9-months, and number of children in the household, main and partner respondent age at birth of cohort child, ethnicity, and highest qualification, family income (banded), OECD poverty level, and housing tenure measured at age three. Where the latter were unavailable at age three, data were fed forward from age 9-months.

### Statistical analysis

#### Software

All descriptive analyses, data transformations, and multiple imputations were conducted in STATA. Main analyses were conducted in Mplus Version 8.7 [46] (see https://osf.io/c6tbh/ for our main analyses Mplus code) and pooled pairwise chi-squared tests were conducted in R using the ‘micombine.chisquared’ function (see https://www.rdocumentation.org/packages/miceadds/versions/3.17-44/topics/micombine.chisquare).

#### Multiple imputation

We multiply imputed missing exposure and outcome variables using fully conditional specifications, with canonical regressions for exposures and predictive mean matching for outcomes given the left-leaning skew of SDQ-TD distributions [47]. We assumed data were missing at random and used multiple imputation by chained equations (MICE) to impute 35 datasets based on the highest fraction of missing information. All variables used within the analysis, as well as interactions between household-level DVA, MH, alcohol use, and drug use (including DVA*MH, DVA*alcohol use, DVA*drug use, MH*alcohol use, MH*drug use, and alcohol use*drug use), were included in the imputation. Interactions between household-level DVA, MH, alcohol use and drug use were included using the ‘just another variable’ approach [45]. This step was essential to ensure that the imputation model was congruent with the analysis method (i.e., LCA; [45]).

Complete case exposure data and multiply imputed data produced similar results and, therefore, the latter are reported from this point onwards (see Supplementary Appendix S2 Tables S2.1 – S2.4 and S3 Tables S3.1 – S3.5, Additional file 1 for missing data patterns and complete case exposure data analyses).

#### Main analyses

First, we created our exposure variable by estimating latent class models using manifest indicators of parental DVA (0 = no, 1 = yes), MH (0 = no, 1 = yes), alcohol use (0 = no, 1 = yes), and drug use (0 = no, 1 = yes) derived using data from age three. We estimated two-, three-, and four-class models. Several criteria were used to assess model fit including scaled relative entropy (0–1, higher entropy scores indicating greater classification certainty), Bayesian Information Criterion (BIC), Akaike Information Criterion (AIC) (lower BIC/AIC scores indicating better fit between the model and observed data), homogeneity in item-response probabilities (indicating the degree of latent class separation), VLMR LRT *p*-value (indicating whether the k model is a better fit to the k-1 model), and interpretability [48, 49]. We re-ran the most appropriate model using the bootstrap likelihood-ratio test (BLRT) to confirm the optimal number of classes [49]. We labelled the resultant latent classes through discussion, taking into account both item-response probabilities and interpretability/theoretical sense-making.

Second, we examined class-specific demographic/contextual profiles using the three-step approach. This involves: (1) estimating the LCA model; (2) retaining information about most likely latent class membership and latent class posterior distributions; and (3) using this information about membership uncertainty to regress latent classes onto demographic/contextual variables in a multinominal regression framework [50]. The three-step procedure in Mplus provides probabilities of demographic/contextual characteristics conditional on membership in a particular latent class [50]. We also report results from pooled pairwise chi-squared tests to indicate whether there are statistically significant differences in the covariate distributions between each latent class.

Third, we fitted a latent growth curve model (LGM) to SDQ-TD scores measured at ages 3–17. Measurement at age three was set as the intercept. We log-transformed the SDQ-TD scores prior to analysis due to a left-leaning skew in the data. We examined intercept-only, linear, and quadratic forms of the LGM and judged model fit using chi-squared (scores > 0.05 indicating good model fit), RSMEA (scores < 0.05 indicating good model fit), CFI and TFLI (scores > 0.95 indicating good model fit), and BIC and AIC scores.

Finally, we related the LCA-derived latent classes to the intercepts, linear, and quadratic terms of the LGM previously estimated. We did this using the case-weight approach whereby: (1) the LCA is estimated; (2) estimated posterior class probabilities are saved as weight variables; and (3) the LGM is estimated for each latent class using case-weight information retained from step two [51]. Latent-class-specific LGM parameters were compared with the original LGM estimated in step four and with one another using Wald chi-squared tests.

Supplementary Appendix S1, Additional file 1 provides a graphical representation of the main analyses.

## Results

**Fig. 1:**
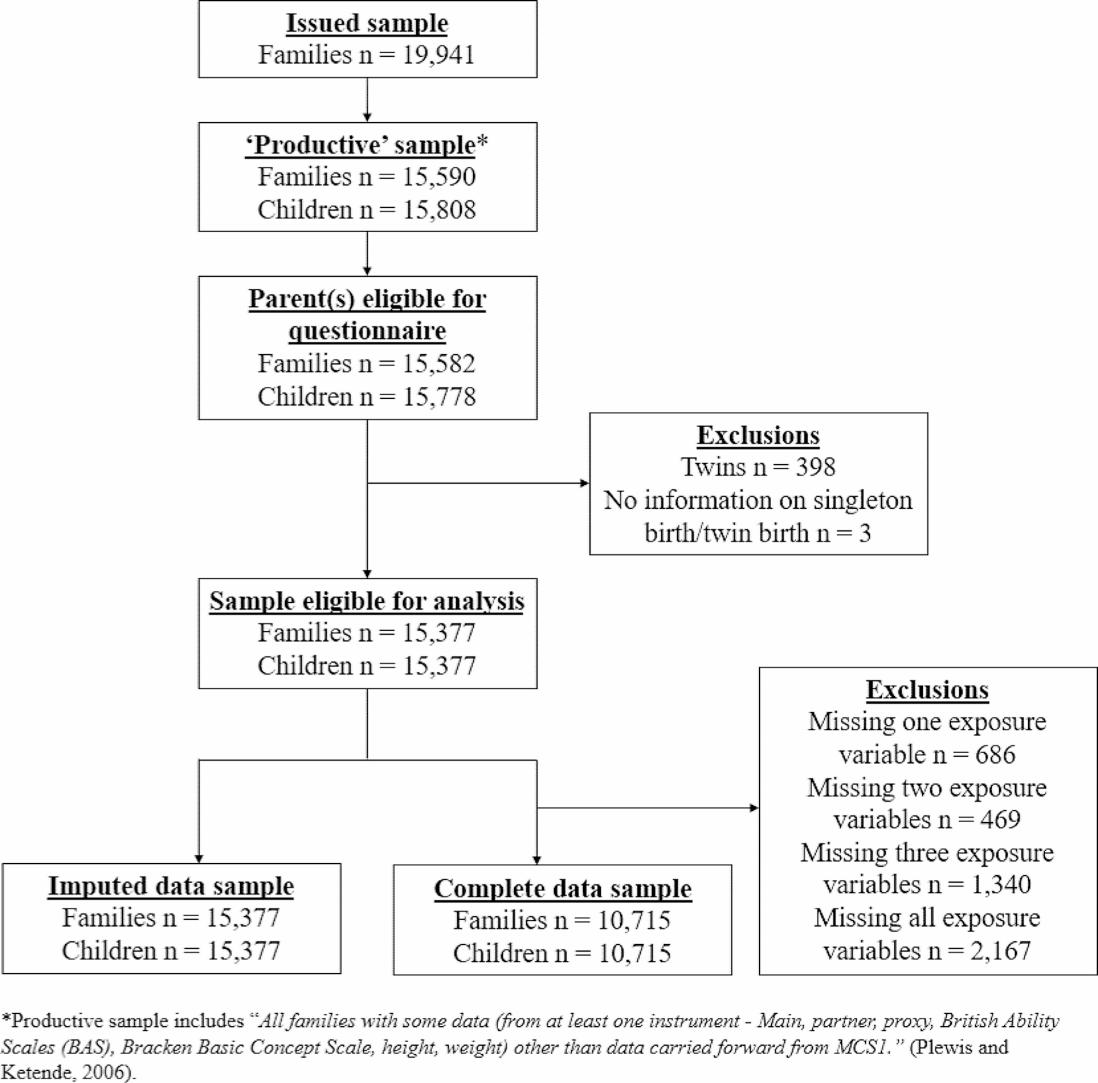
Participant flow through study

Figure 1 illustrates the flow of participants through the study. At child age three, 9.8% (*n* = 1,507) of families reported parental DVA, 4.3% (*n* = 661) reported poor parental MH, 12.8% (*n* = 1,968) reported parental alcohol use, and 9.7% (*n* = 1,492) reported parental drug use (Table 2). Mean SDQ-TD scores across sweeps ranged from 7.5 (SE = 0.04) to 9.8 (SE = 0.04).

**Table 2:** Estimated response probabilities for exposure variables and mean and SEs for outcome variables (multiply imputed data)

**Exposure variables**	**Imputed sample**			
	**Main respondent** **(** ***n*** ** = 15,377)**	**Partner respondent** **(** ***n*** ** = 12,677)**		Household* **(** ***n*** ** = 15,377)**
**Parental DVA (physical violence)**				
No	14,654 (95.3%)	11,599 (91.5%)		13,870 (90.2%)
Yes	723 (4.7%)	1,078 (8.5%)		1,507 (9.8%)
**Parental MH**				
No	14,885 (96.8%)	12,500 (98.6%)		14,716 (95.7%)
Yes	492 (3.2%)	177 (1.4%)		661 (4.3%)
**Parental alcohol use (frequency)**				
No	14,547 (94.6%)	11,130 (87.8%)		13,409 (87.2%)
Yes	830 (5.4%)	1,547 (12.2%)		1,968 (12.8%)
**Parental drug use**				
No	14,747 (95.9%)	11,599 (91.5%)		13,885 (90.3%)
Yes	630 (4.1%)	1,078 (8.5%)		1,492 (9.7%)
**Outcome variables**	**Cohort child (** ***n*** ** = 15,** ** 377)**			
	**Non-log transformed**		**Log transformed**	
**SDQ total difficulties - Sweep 2 (child 3-years-old)**				
Overall - Mean (SE)	9.8 (0.04)		2.24 (0.005)	
Boys - Mean (SE)	10.3 (0.06)		2.29 (0.006)	
Girls - Mean (SE)	9.3 (0.06)		2.18 (0.006)	
**SDQ total difficulties - Sweep 3 (child 5-years-old)**				
Overall - Mean (SE)	7.5 (0.04)		1.94 (0.006)	
Boys - Mean (SE)	8.0 (0.06)		2.01 (0.007)	
Girls - Mean (SE)	6.9 (0.06)		1.87 (0.008)	
**SDQ total difficulties - Sweep 4 (child 7-years-old)**				
Overall - Mean (SE)	7.6 (0.05)		1.93 (0.006)	
Boys - Mean (SE)	8.3 (0.07)		2.02 (0.008)	
Girls - Mean (SE)	6.9 (0.06)		1.84 (0.009)	
**SDQ total difficulties - Sweep 5 (child 11-years-old)**				
Overall - Mean (SE)	7.8 (0.05)		1.94 (0.006)	
Boys - Mean (SE)	8.5 (0.07)		2.02 (0.008)	
Girls - Mean (SE)	7.2 (0.07)		1.86 (0.009)	
**SDQ total difficulties - Sweep 6 (child 14-years-old)**				
Overall - Mean (SE)	8.3 (0.05)		2.0 (0.006)	
Boys - Mean (SE)	8.6 (0.08)		2.03 (0.009)	
Girls - Mean (SE)	8.0 (0.07)		1.97 (0.01)	
**SDQ total difficulties - Sweep 7 (child 17-years-old)**				
Overall - Mean (SE)	7.8 (0.05)		1.92 (0.007)	
Boys - Mean (SE)	7.8 (0.08)		1.92 (0.01)	
Girls - Mean (SE)	7.7 (0.08)		1.92 (0.01)	

* Only household-level DVA, MH, alcohol use, and drug use variables used as manifest indicators in LCA. **NB.** All *ns* reported are estimations based on the 35 imputed datasets

### Latent class analysis

We estimated two, three, and four-class latent class models using complete case exposure data (Supplementary Appendix S3, Additional file 1). The three-class model provided the best fit in terms of entropy (0.74), AIC, and c-BIC, as well as demonstrating clearer class separation and theoretical sense in classes as compared to the two- and four-class models (Table 3). The three-class solution also displayed a significant VLMR *p*-value. Both the two-class and four-class solutions displayed low levels of entropy (0.57 and 0.37, respectively), and the four-class solution also suffered from low classification accuracy and unclear class separation, complicating interpretation. Although the two-class model displayed a lower BIC score than the three-class solution, the difference between the two was minimal (< 9.0) (Table 3).

**Table 3:** Latent class analysis fit indices for estimated models and final three-class model (complete case exposure data and multiply imputed data)

Model fit	Number of latent classes estimated*			Final model
	2 classes	3 classes	4 classes	3 classes
Entropy	0.566	**0**.**748**	0.374	**0**.**758**
AIC	25677.21	**25653.95**	25663.03	36269.93
BIC	**25742.72**	25755.86	25801.34	36376.90
c-BIC	25714.12	**25711.37**	25740.96	36332.41
VLMR LRT *p* value	*p* < 0.001	*p* < 0.001	*p* = 0.8342	-
BLRT *p* value	-	***p*** ** < 0.001**	-	-

*We assessed LCA model fit was using complete case exposure data (*n* = 10,715) and re-ran the selected model using imputed data (*n* = 15,377; ‘final model’). **NB.** AIC = Akaike Information Criterion (AIC); BIC = Bayesian Information Criterion; c-BIC = sample size adjusted BIC; VLMR LRT = Vuong-Lo-Mendell-Rubin Likelihood Ratio Test; BLRT = Bootstrap Likelihood-Ratio Test

Given the judged fit of the three-class model, we ran a BLRT to confirm the model fit, which was significant (*p* < 0.001), so the three-class model was selected and re-run using the imputed data (see Table 3, ‘final model’). Based on the item-response probabilities for DVA, MH, alcohol use, and drug use in each latent class within the imputed data (Table 4), we labelled the latent classes as follows:

**Table 4:** Latent classes, item-responses, and membership probabilities for final three-class model (multiply imputed data)

Final model three-class model			
LCA indicators	Latent class item-response probabilities		
	Latent Class 1 “High-frequency alcohol use”	Latent Class 2 “Elevated adversity”	Latent Class 3 “Low-level adversity”
**Parental DVA (physical violence)**			
No	0.914	0.528	0.949
Yes	0.086	0.472	0.051
**Parental MH**			
No	0.993	0.835	0.965
Yes	0.007	0.165	0.035
**Parental alcohol use (frequency)**			
No	0	0.785	0.977
Yes	1	0.215	0.023
**Parental drug use**			
No	0.885	0.562	0.945
Yes	0.115	0.438	0.055
**Proportions of sample**	11.9%	3.5%	84.6%

#### Class 1: High-frequency alcohol use

This class comprised 11.9% of families, who reported drinking alcohol 5–6 times per week or everyday (100%). Families in this class were unlikely to be experiencing DVA (8.6%), MH (0.7%), or drug use (11.5%).

#### Class 2: Elevated adversity

This class was the smallest class (3.5%). Families were more likely to be experiencing DVA (47.2%), MH (16.5%), or drug use (43.8%) as compared to the other two classes. They were also more likely to be experiencing high-frequency alcohol use (21.5%) as compared to the *low-level adversity* class.

#### Class 3: Low-level adversity

This class was the largest class (84.6%). Families in this class displayed low levels of DVA (5.6%), MH (3.3%), alcohol use (2.5%), and drug use (5.5%).

### Demographic/contextual profiles of latent classes

Latent-class-specific demographic/contextual profiles are presented in Table 5, along with details of the pooled chi-squared tests between each latent class for the multiply imputed data.

**Table 5:** Demographic/contextual profiles of each latent class in final three-class model (multiply imputed data)

Demographic and contextual variables	Conditional probabilities given latent class			Differences between latent classes^		
	Latent Class 1 “High-frequency alcohol use”	Latent Class 2 “Elevated adversity”	Latent Class 3 “Low-level adversity”	Class 1 vs. Class 2	Class 1 vs. Class 3	Class 2 vs. Class 3
**Child sex**						
Male	52.4%	52.7%	50.8%	Pooled chi-squared F(1) = 0.097 *p*-value = 0.756	Pooled chi-squared F(1) = 0.64 *p*-value = 0.424	Pooled chi-squared F(1) = 0.503 *p*-value = 0.478
Female	47.6%	47.3%	49.2%			
**Number of children in household (including cohort child)**						
One	19.8%	22.0%	26.5%	Pooled chi-squared F(4) = 3.34 *p*-value = 0.01*	Pooled chi-squared F(4) = 12.737 *p*-value = < 0.001*	Pooled chi-squared F(4) = 0.556 *p*-value = 0.695
Two	54.9%	44.2%	43.6%			
Three	18.9%	20.8%	19.0%			
Four	5.2%	8.8%	7.4%			
Five or more	2.2%	4.3%	3.5%			
**Main respondent age at birth**						
< 18 years	0.0%	3.2%	2.6%	Pooled chi-squared F(5) = 56.853 *p*-value = < 0.001*	Pooled chi-squared F(5) = 22.578 *p*-value = < 0.001*	Pooled chi-squared F(5) = 10.881 *p*-value = < 0.001*
18–25 years	1.6%	46.2%	27.4%			
26–30 years	26.2%	29.5%	30.3%			
31–35 years	43.9%	17.5%	27.2%			
36–40 years	24.5%	3.3%	10.7%			
> 40 years	3.8%	0.3%	1.7%			
**Partner respondent age at birth**†						
< 18 years	0.0%	1.4%	0.4%	Pooled chi-squared F(5) = 58.603 *p*-value = < 0.001*	Pooled chi-squared F(5) = 36.959 *p*-value = < 0.001*	Pooled chi-squared F(5) = 18.433 *p*-value = < 0.001*
18–25 years	0.2%	29.5%	13.1%			
26–30 years	13.6%	32.3%	26.1%			
31–35 years	37.9%	22.4%	34.0%			
36–40 years	30.7%	12.2%	18.3%			
> 40 years	17.7%	2.3%	8.1%			
**Main respondent ethnicity**						
White	99.4%	91.9%	82.5%	Pooled chi-squared F(1) = 12.663 *p*-value = < 0.001*	Pooled chi-squared F(1) = 5.765 *p*-value = < 0.022	Pooled chi-squared F(1) = 18.038 *p*-value = < 0.001*
Ethnic minority	0.6%	8.1%	17.5%			
**Partner respondent ethnicity**†						
White	100.0%	88.7%	82.2%	Pooled chi-squared F(1) = 25.865 *p*-value = < 0.001*	Pooled chi-squared F(1) = 25.642 *p*-value = < 0.001*	Pooled chi-squared F(1) = 9.249 *p*-value = < 0.003*
Ethnic minority	0.0%	11.3%	17.8%			
**Main respondent highest qualification**						
Higher degree	8.6%	2.3%	3.6%	Pooled chi-squared F(7) = 19.816 *p*-value = < 0.001*	Pooled chi-squared F(7) = 49.259 *p*-value = < 0.001*	Pooled chi-squared F(7) = 2.077 *p*-value = 0.043
First degree	29.1%	7.1%	12.5%			
Diploma	12.5%	8.7%	9.3%			
A/AS/L Level	13.8%	8.6%	9.3%			
GCSE A-C	26.8%	35.4%	33.1%			
GCSE D-G	4.5%	13.3%	10.7%			
Other	0.8%	1.7%	3.3%			
None listed	3.8%	22.8%	18.2%			
**Partner respondent highest qualification**†						
Higher degree	10.9%	1.3%	6.1%	Pooled chi-squared F(7) = 19.784 *p*-value = < 0.001*	Pooled chi-squared F(7) = 26.383 *p*-value = < 0.001*	Pooled chi-squared F(7) = 6.286 *p*-value = < 0.001*
First degree	25.9%	5.8%	14.1%			
Diploma	10.0%	6.2%	8.9%			
A/AS/L Level	9.7%	6.8%	7.0%			
GCSE A-C	29.9%	33.6%	29.5%			
GCSE D-G	5.3%	14.8%	9.9%			
Other	0.7%	3.7%	3.6%			
None listed	7.7%	27.9%	20.8%			
**Joint annual income**						
£0 - £3300	0.8%	5.4%	4.2%	Pooled chi-squared F(5) = 22.708 *p*-value = < 0.001*	Pooled chi-squared F(5) = 0.624 *p*-value = 0.682	Pooled chi-squared F(5) = 4.823 *p*-value = < 0.001*
£3300 - £11,000	0.0%	28.6%	21.5%			
£11,000 - £22,000	17.4%	36.9%	29.9%			
£22,000 - £33,000	27.5%	18.9%	21.3%			
£33,000 - £55,000	34.6%	7.6%	17.6%			
£55,000+	19.7%	2.6%	5.5%			
**Poverty level**						
Above 60% poverty level	99.0%	50.5%	65.7%	Pooled chi-squared F(1) = 48.175 *p*-value = < 0.001*	Pooled chi-squared F(1) = 472.704 *p*-value = < 0.001*	Pooled chi-squared F(1) = 12.217 *p*-value = < 0.001*
Below 60% poverty level	1.0%	49.5%	34.3%			
**Housing tenure**						
Own	96.1%	33.0%	65.5%	Pooled chi-squared F(3) = 84.89 *p*-value = < 0.001*	Pooled chi-squared F(3) = 116.835 *p*-value = < 0.001*	Pooled chi-squared F(3) = 26.39 *p*-value = < 0.001*
Rent council / housing association	0.6%	47.9%	24.0%			
Rent private	2.9%	16.0%	6.5%			
Living with parents and other	0.5%	3.1%	3.9%			

† Partner respondents *n* = 12,677. ^ Differences between classes estimated using pooled pairwise chi-squared F statistics across 35 imputed datasets. * Statistically significant *p*-value. Significance was determined based on an adjusted alpha value of 0.017 (i.e., 0.05/3) to account for multiple testing

#### Child sex

Child sex did not significantly differ by latent class.

#### Number of children in household

The high-frequency alcohol use class displayed significantly higher probabilities of having two children in the household and lower probabilities of having one, four, or five or more, as compared to the other two classes.

#### Main and partner respondent age at birth

Main and partner respondents in the *elevated adversity* class had higher probabilities of being younger, whereas those in the *high-frequency alcohol use* class had higher probabilities of being older. Most main and partner respondents in the *low-level adversity* class were aged between 26 and 35 years.

#### Main and partner respondent ethnicity

Main respondents in the *elevated adversity* class had higher probabilities of being White and lower probabilities of being in an Ethnic Minority group as compared to the *low-level adversity* class. When compared to the *high-frequency alcohol use* class, main respondents in the *elevated adversity* class had lower probabilities of being White and higher probabilities of being in an Ethnic Minority group. For partner respondents, there were higher probabilities of being White in the *high-frequency alcohol use* class as compared to the *low-level adversity* and *elevated adversity* class. Consequently, there were higher probabilities of partner respondents being in an Ethnic Minority group in the *low-level adversity* class as compared to the *multiple-adversity* and *high-frequency alcohol use* class.

#### Main and partner respondent qualifications

Main and partner respondents in the *high-frequency alcohol use* class had higher probabilities of continuing their education beyond secondary school (i.e., beyond age 16) compared to those in the *elevated adversity* class who had higher probabilities of having no qualifications or GCSEs only. Main and partner respondents in the *low-level adversity* class were more mixed. There were no significant differences between main respondent qualifications in the *elevated adversity* class as compared to the *low-level adversity* class.

#### Joint annual income

Respondents in the *elevated adversity* class had higher probabilities of having a lower joint annual income as compared to the *high-frequency alcohol use* class and the *low-level adversity* class.

#### Poverty

Almost all respondents in the *high-frequency alcohol use* class were above the poverty line whereas almost half of respondents in the *elevated adversity* class were below the poverty line. For the *low-level adversity* class, families had higher probabilities of being above the poverty line.

#### Housing tenure

Most respondents in the *high-frequency alcohol use* class owned their own house as did most respondents in the *low-level adversity* class, although to a lesser extent. The *elevated adversity* class displayed higher probabilities of renting from the local authority or a housing association and renting privately as compared to the other two classes.

### Relation to child mental health

Table 2 shows the estimated mean SDQ-TD scores at each timepoint. For the overall, unconditional model, the quadratic terms provided the best model fit (RMSEA = 0.116, CFI = 0.910, χ2 (df12) = 2492.3, *p* < 0.001; Table 6). The mean intercept for the log-transformed SDQ-TD score was 2.163 (*p* < 0.001), the mean linear slope was −0.057 (*p* < 0.001), and the mean quadratic slope was 0.003 (*p* < 0.001). Children’s MH gradually improved from 3 to 11 years at which point MH began to gradually deteriorate from 11 to 17 years. There was significant variability in the intercept, linear slope, and quadratic slope indicating children’s MH differed in terms of the intercept and change across time.

**Table 6:** Unconditional and latent-class-specific latent growth curve models (multiply imputed data)

	**Overall latent growth curve models**							
	**Model 1**		**Model 2**		**Model 3**		**Model 4**	
	**Estimate**	**95% CIs**	**Estimate**	**95% CIs**	**Estimate**	**95% CIs**	**Estimate**	**95% CIs**
**Mean**								
Intercept	1.988*	(1.979, 1.997)	2.080*	(2.07, 2.088)	2.163*	(2.153, 2.172)	2.218*	(2.205, 2.232)
Linear slope	-	-	-0.012*	(-0.013, -0.011)	-0.057*	(-0.06, -0.055)	-0.047*	(-0.051, -0.043)
Quadratic slope	-	-	-	-	0.003*	(0.003, 0.003)	0.002*	(0.002, 0.002)
**Variance**								
Intercept	0.273*	(0.265, 0.281)	0.249*	(0.240, 0.257)	0.208*	(0.2, 0.216)	0.205*	(0.197, 0.213)
Linear slope	-	-	0.001*	(0.001, 0.001)	0.007*	(0.007, 0.008)	0.007*	(0.006, 0.008)
Quadratic slope	-	-	-	-	<0.001*	(<0.001, <0.001)	<0.001*	(<0.001, <0.001)
**Child sex (girls)**								
Intercept	-	-	-	-	-	-	-0.114*	(-0.132, -0.096)
Linear slope	-	-	-	-	-	-	-0.021*	(-0.027, -0.015)
Quadratic slope	-	-	-	-	-	-	0.002*	(0.002, 0.004)
**Fit indices**								
χ2 (df)	7751.757 (19)*		4573.217 (16)*		**2492.373** (12)*		2545.436 (15)*	
RMSEA (CI)	0.163 (0.160–0.166)		0.136 (0.133–0.139)		0.116 (0.112–0.120)		**0.105** (0.101–0.108)	
CFI	0.721		0.835		0.910		**0.913**	
SRMR	0.292		0.188		0.084		**0.076**	
	**Latent-class-specific models**							
	**Latent class 1** ***“High-frequency alcohol use”***			**Latent class 2** ***“Elevated adversity”***		**Latent class 3** ***“Low-level adversity”***		
	**Estimate**		**95% CIs**	**Estimate**	**95% CIs**	**Estimate**	**95% CIs**	
**Mean**								
	2.068*		(2.028, 2.109)	2.274*	(2.248, 2.301)	2.228*	(2.214, 2.242)	
	-0.045*		(-0.057, -0.032)	-0.040*	(-0.048, -0.033)	-0.048*	(-0.053, -0.044)	
	0.002*		(0.001, 0.003)	0.002*	(0.001, 0.002)	0.002*	(0.002, 0.003)	
**Variance**								
	0.188*		(0.163, 0.214)	0.202*	(0.185, 0.219)	0.204*	(0.195, 0.213)	
	0.006*		(0.003, 0.008)	0.007*	(0.005, 0.008)	0.007*	(0.007, 0.008)	
	<0.001*		(<0.001, <0.001)	<0.001*	(<0.001, <0.001)	<0.001*	(<0.001, <0.001)	
**Child sex (girls)**								
	-0.111*		(-0.166, -0.056)	-0.096*	(-0.133, -0.06)	-0.117*	(-0.136, -0.097)	
	-0.024†		(-0.042, -0.007)	-0.022*	(-0.032, -0.012)	-0.021*	(-0.027, 0.014)	
	0.002*		(0.001, 0.004)	0.002*	(0.001, 0.003)	0.002*	(0.004, 0.008)	

* *p *≤ 0.001 † *p *< 0.05. Model 1 = Unconditional model, random intercept only (fixed slope); Model 2 = Unconditional model, random intercept and linear slope; Model 3 = Unconditional model, random intercept, linear, and quadratic slope; Model 4 = Conditional model (including child sex as fixed effect covariate), random intercept, linear, and quadratic slope, obtained as main coefficients. See Supplementary Appendix S4, Additional file 1 for covariance matrix

**Fig. 2:**
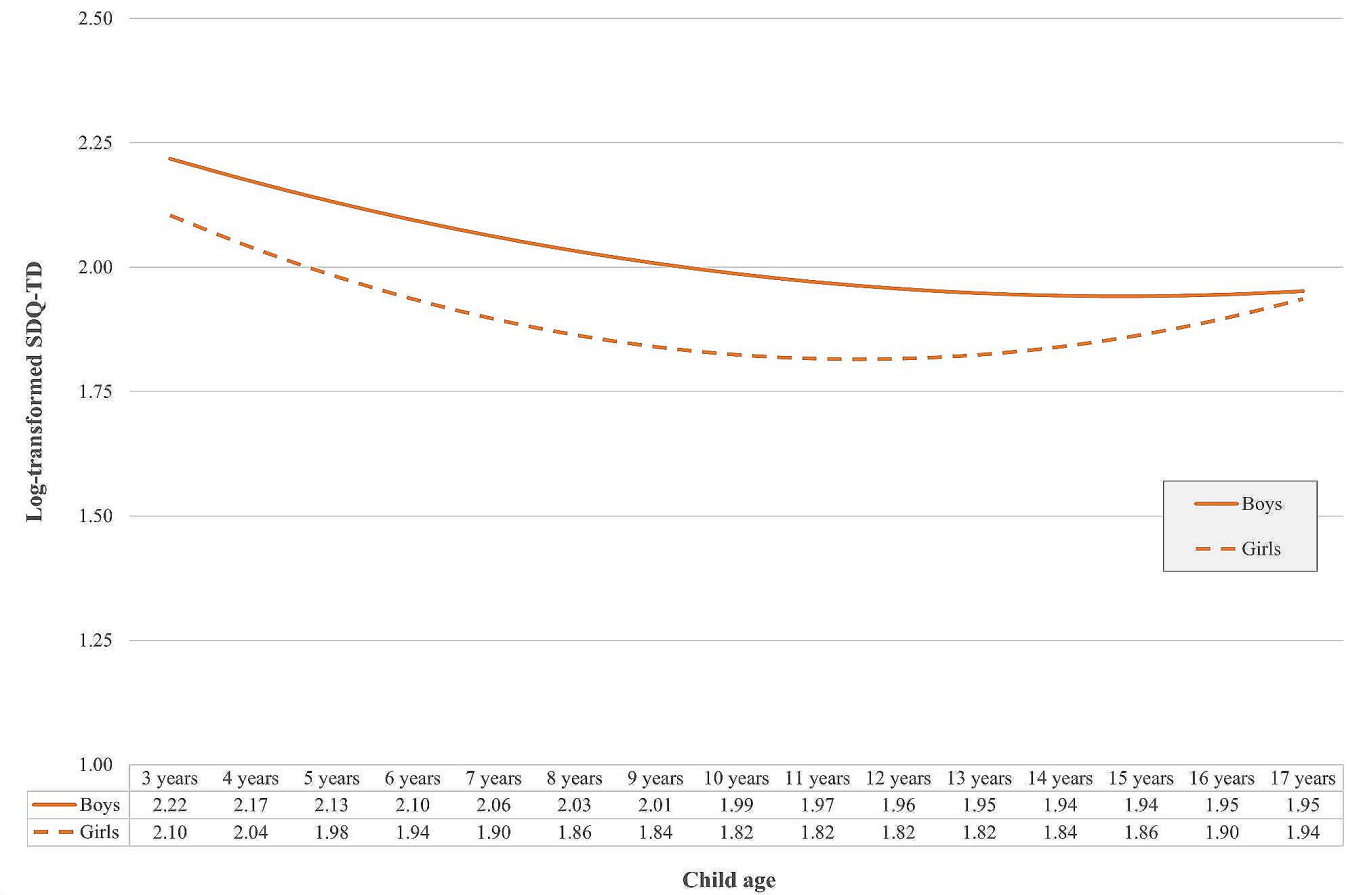
Growth curves for boys and girls (multiply imputed data)

Given known gender differences in MH, we added child sex as a time-invariant covariate in a conditional model of child MH trajectories. This conditional model appeared to provide a marginally better fit as compared to the unconditional model (RMSEA = 0.105, CFI = 0.913, χ2 (df15) = 2545.436, *p* < 0.001; Table 6), although the fit indices were still not optimal. There was a significant effect of child sex on the mean intercept, linear slope, and quadratic slope. Figure 2 displays the growth trajectories for boys and girls, separately, along with the marginal mean predictions for boys and girls at specific sensitive ages (min, median, max). Girls were more likely to have a better mean intercept SDQ-TD score than boys (estimate = -0.114; *p* < 0.001). Boys’ MH gradually improved from 3 to 14 years after which they plateaued, with a slight increase at 17 years (linear slope estimate = -0.047, *p* < 0.001; quadratic slope estimate = 0.002, *p* < 0.001). Girls’ MH was marked by a steeper improvement in scores from 3 to 11 years, at which point MH gradually began to deteriorate (linear slope estimate = -0.021; *p* < 0.001; quadratic slope estimate = 0.002; *p* < 0.001).

**Fig. 3:**
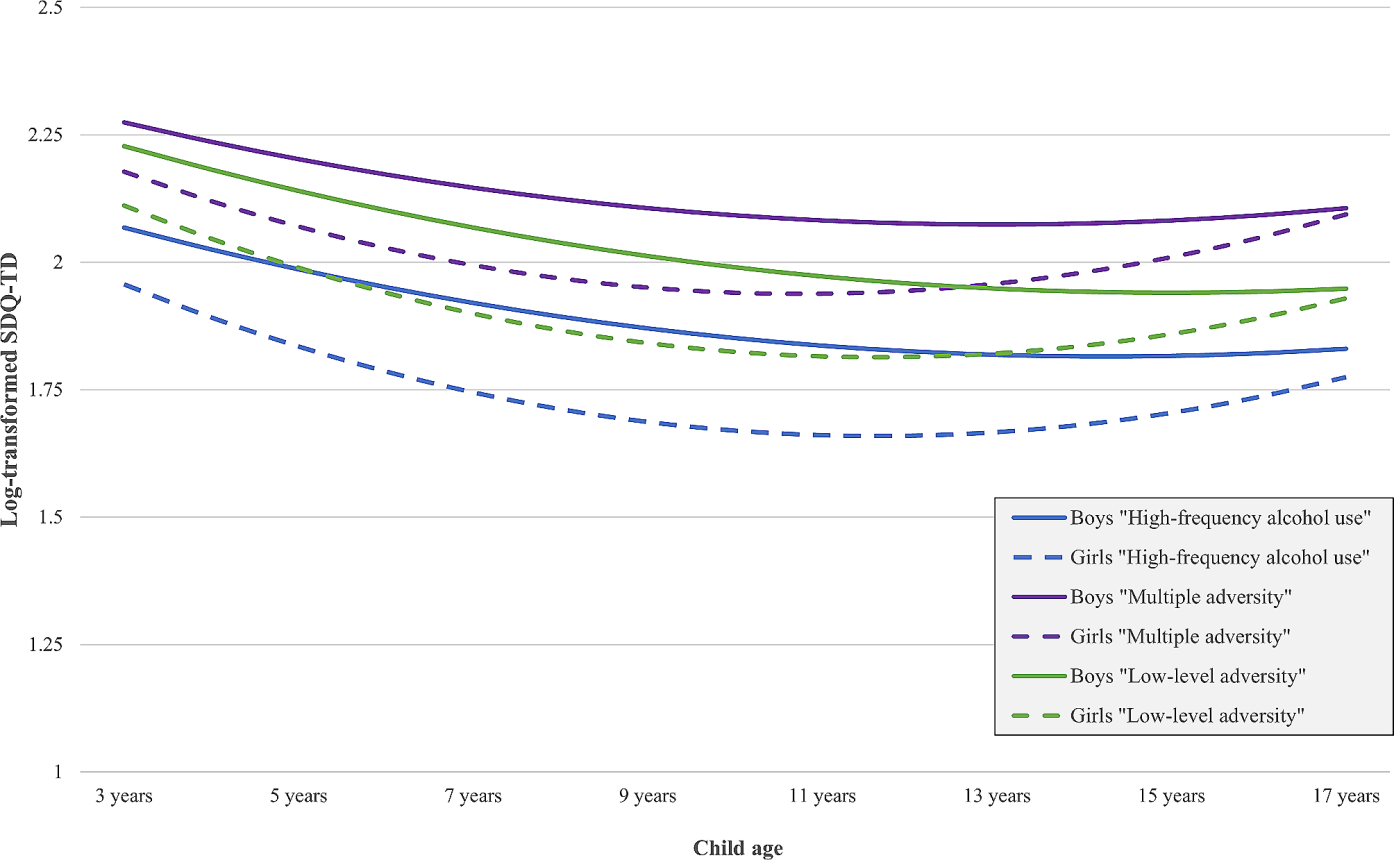
Latent-class-specific growth curves for boys and girls (multiply imputed data)

Finally, we examined latent-class-specific child MH trajectories controlling for child sex (Table 6; Fig. 3). Latent-class-specific trajectories followed a similar growth pattern (as indicated by similar linear and quadratic slope estimates for each latent class). However, the intercepts significantly differed between latent classes (Table 6; Supplementary Appendix S4, Additional file 1). Children in the *elevated adversity* class displayed the highest SDQ-TD scores at age three (intercept = 2.274; *p* < 0.001), followed by the *low-level adversity* (intercept = 2.228; *p* < 0.001), and *high-frequency alcohol* class (intercept = 2.068; *p* < 0.001). All three latent-class-specific trajectories demonstrated significant variability in intercepts, linear slopes, and quadratic slopes, indicating children’s MH within each latent class differed in terms of the intercept and change across time.

## Discussion

We examined how parental DVA, MH, alcohol use, and drug use cluster during early childhood in the MCS, the demographic/contextual profiles of these clusters, and how these clusters relate to children’s MH trajectories. We identified three latent clusters: *high-frequency alcohol use*, *elevated adversity*, and *low-level adversity*. These clusters were related to distinct demographic/contextual profiles and child MH profiles.

At child age three, 3.5% of families were experiencing *elevated adversity* which concurs with studies estimating the prevalence of parental DVA, MH, and SU in the UK and extends previous individual/cumulative ACE work [8]. Our *elevated adversity* cluster was similar to that identified in a sample of families referred to statutory Children’s Social Care in England and general population studies where *lifetime* DVA, MH, and SU cluster alongside other ACEs [26, 28]. However, it is one of few to find parental DVA, MH, and SU cluster as a triad alone. This may be due to differences in the time point examined, our consideration of both main and partner responses, or our inclusion of parental drug use which features clearly in our *elevated adversity* class but has been excluded from other studies [31]. The item-response probabilities of parental MH in this cluster were notably low, which is interesting given the established bi-directional relationship between MH and DVA/SU and predominance of MH as an ACE in other studies [16, 26, 31, 52]. We used a cut-off point for poor MH to reflect clinical caseness which may explain this finding. Equally, general population studies may not be capturing data from families experiencing the greatest challenges.

Families in the *elevated adversity* class tended to be White (although to a lesser extent than the high frequency alcohol use class), younger parents from low-socioeconomic backgrounds, highlighting key potential demographic/contextual targets for future intervention. We did not include poverty in our ACE cluster given that prevention and early intervention in relation to poverty and DVA, MH, and SU have different policy and practice implications. Poverty is often considered a social policy issue while DVA, MH, and SU are considered health and social care practice concerns. However, our study emphasises that poverty cannot be ignored when considering clustered parental DVA, MH, and SU; almost *half* of families experiencing this cluster fell below the poverty line. While being mindful not to conflate the two [53], there is a clear link between poverty and individual/cumulative ACEs as well as clustered adversity [26, 54]. Thus, interventions aiming to prevent/reduce clustered ACEs should actively target *economic* risk factors, as well as individual/family-level risk factors. Interventions for parental DVA, MH, and SU often target low-income families or areas but rarely directly address poverty, even though socioeconomic support alone has been shown to reduce the prevalence of ACEs [55]. Future work would benefit from reframing ACE clusters as syndemic issues; not only examining how ACEs cluster or amplify one another but also exploring population-level social, economic, environmental, and political determinants which may be amenable to policy/system-level intervention [56].

The fact that our *elevated adversity* class was associated with worse child MH at age three (and subsequently, across time) serves to validate the policy focus on parental DVA, MH, and SU where this has been previously questioned [30]. Intervening early to support children and families with complex needs may minimise the potential disadvantage children face in terms of their MH. Early years education settings are likely to be useful sites for prevention of child MH problems. Future research should explore whether the impact of parental DVA, MH, and SU can be detected even earlier in a child’s development, as well elucidating the causal pathway between these clustered ACEs and child MH. This should be guided by frameworks such as Family Stress Theory which also consider the role poverty might play [57].

Unsurprisingly, there were high levels of variability in our latent-class-specific trajectories of child MH, which may be influenced by mediators/moderators, severity, or persistence of clustered-risk. Future work should examine: (1) more nuanced, differential latent trajectories associated with exposure to clustered parental DVA, MH, and SU and associated risk and protective factors, and (2) whether changes or stability in latent class membership over time might be associated with differential child MH outcomes.

Our study benefits from several strengths, including the use of a large nationally representative sample, prospective measurement of parental DVA, MH, and SU, consistent measurement of child MH, and pre-registered analysis plans. However, we need to consider its limitations. First, our measurement of parental DVA was restricted to a single-item on physical violence, the only available variable as with many large cohort studies. DVA can include physical, sexual, emotional, controlling or coercive, and economic abuse, all of which are essential to consider given the impact they can have on families [18]. Second, our measure of alcohol use was limited to frequency, as in other studies [31]. Although indicative of problematic use, our study suggests this is a poor ACE measure. Considering the quantity of alcohol use and impact on daily living would be preferable. Third, we were unable to consider severity of DVA, MH, and SU which may influence the impact on child outcomes [32]. Fourth, measures of DVA, MH, and SU relied on self-report which could be prone to social-desirability bias. This may be particularly pertinent for ethnic minority groups due to cultural stigma associated with DVA, MH, or SU. However, the prevalence of these issues appeared similar to other studies and, situated in the context of other questions, social-desirability bias is likely to explain a small amount of variance in responses [58]. Fifth, both our exposure variables and outcome variables rely on parent-reported measures, increasing the risk of information bias. Finally, while the fit indices of our overall and conditional LGM were sufficient, they were not optimal, meaning there is room for improvement to enhance the robustness and replicability of the model findings.

## Conclusions

Improving support for families who experience co-occurring parental DVA, MH, and SU is a key priority for policy and practice. Our study validates this focus, finding that parental DVA, MH, and SU cluster during early child development and can have a negative and persistent impact on children’s MH as young as three-years of age. Our findings suggest preventing and responding to this clustered risk requires an early, multi-faceted response that addresses all these ACEs in combination as well as the socio-economic determinants that drive them.

### Electronic supplementary material

Below is the link to the electronic supplementary material.

Supplementary Material 1

## Data Availability

This study uses data from the MCS. MCS data can be found and accessed via the UK Data Service: https://ukdataservice.ac.uk/.

## Abbreviations

AIC: Akaike information criterion
ACE: Adverse childhood experience
BIC: Bayesian information criterion
BLRT: Bootstrap likelihood-ratio test
c-BIC: Sample size adjusted Bayesian information criterion
DVA: Domestic violence and abuse
LCA: Latent class analysis
LGM: Latent growth curve model
MCS: Millennium Cohort Study
MH: Mental ill-health
MICE: Multiple imputation by chained equations
OECD: Organisation for Economic Co-operation and Development
SDQ: Strengths and Difficulties Questionnaire
SDQ-TD: Strengths and Difficulties Questionnaire Total Difficulties Score
SU: Substance misuse
VLMR LRT: Vuong-Lo-Mendell-Rubin adjusted likelihood-ratio test
