# Supplementary material for: Parental domestic violence and abuse, mental ill-health, and substance misuse and the impact on child mental health: a secondary data analysis using the UK Millennium Cohort Study
Source: BMC Public Health. 2024 Aug 26;24:2310. doi: 10.1186/s12889-024-19694-1 (PMC11346203; doi:10.1186/s12889-024-19694-1)
Supplement: Supplementary file 1 — Supplementary Material 1 [file 12889_2024_19694_MOESM1_ESM.docx]

**Additional file 1**

Table of Contents

[Appendix S1. Additional information on methods and analyses 2](#_Toc173919190)

[S1.1. Detail and rationale for latent indicators and outcome variable 2](#_Toc173919191)

[Figure S1.2. Graphical representation of main analyses 4](#_Toc173919192)

[Appendix S2. Missing data patterns and comparisons between complete and multiply imputed data 5](#_Toc173919193)

[Table S2.1. Distribution of completeness of household exposure variables at sweep 2 (child three years of age) 5](#_Toc173919194)

[Table S2.2. Distribution of completeness of SDQ outcome variables from sweep 2–7 (child 3–17 years of age) 6](#_Toc173919195)

[Table S2.3. Responses rates and percentages for exposure variables and response rates and means and SDs for outcome variables for total sample, complete exposure data sample, incomplete exposure data sample, and imputed sample 7](#_Toc173919196)

[Table S2.4. Demographic/contextual characteristics for total sample, complete exposure data sample, incomplete exposure data sample, and imputed sample 9](#_Toc173919197)

[Appendix S3. Complete exposure data case analyses (n=10,715) 13](#_Toc173919198)

[Table S3.1. Latent class analysis fit indices (complete exposure data) 13](#_Toc173919199)

[Table S3.2. Latent classes, item-responses, and membership probabilities for each estimated latent class model (complete exposure data) 13](#_Toc173919200)

[Table S3.3. Demographic/contextual profiles of each latent class in selected three-class model (complete exposure data) 16](#_Toc173919201)

[Table S3.4. Unconditional, conditional, and latent-class-specific latent growth curve models (complete exposure data) 21](#_Toc173919202)

[Table S3.5. Wald test of parameter constraints for overall, intercept, and linear and quadratic slope (complete exposure data) 23](#_Toc173919203)

[Appendix S4. Multiply imputed data analyses (n=15,377) 24](#_Toc173919204)

[Table S4.1. Covariance matrix for unconditional and latent-class-specific latent growth curve models (multiply imputed data) 24](#_Toc173919205)

[Table S4.2. Wald test of parameter constraints for overall, intercept, and linear and quadratic slope (multiply imputed data) 25](#_Toc173919206)

[References 26](#_Toc173919207)

# Appendix S1. Additional information on methods and analyses

## S1.1. Detail and rationale for latent indicators and outcome variable

*Detail and rationale for latent indicator categorisations*

Each latent indicator was defined as follows. Prescence of parental DVA was operationalised as any physical violence experienced by main or partner respondent in their relationship. We categorised parental DVA this way in recognition of the fact that regardless of who is perpetrating physical violence, any DVA in the household is likely to impact child outcomes. Prescence of parental MH was operationalised as scores ≥13 on the Kessler Distress Scale-6 (<https://www.hcp.med.harvard.edu/ncs/k6_scales.php>) by main or partner respondent. We categorised parental MH in this way in keeping with the MCS and because such scores have been shown to be indicative of high levels of common MH which may impact child outcomes (1). Prescence of parental alcohol use was operationalised as high-frequency drinking (every day or 5-6 times per week) of main or partner respondent. We categorised parental alcohol use in this way in recognition of the fact that high-frequency drinking may be indicative of problematic alcohol use and to be in keeping with other research in this area (2, 3). Finally, presence of parental drug use was operationalised as any drug use by main or partner respondents. We categorised parental drug use in this way given that drug use is illegal within the UK and is likely to have detrimental impacts on children. This categorisation is in keeping with previous literature (4).

*Detail and rationale for outcome variable*

The SDQ is a widely used measure of child mental health which demonstrates good reliability and validity, and has shown discriminative ability to detect psychiatric disorders (including conduct-oppositional, hyperactivity, depression and some anxiety disorders) within a large community sample of children in Britain (5, 6). Although it is ideal to combine data from multiple informants to improve the discriminative power of the SDQ, when using a single informant, the parent version of the SDQ is the best at identifying emotional disorders (6). Consequently, in combination with the fact that parent-reported SDQ was measured consistently throughout the MCS sweeps, we used parent-reported SDQ as our outcome variable.

##
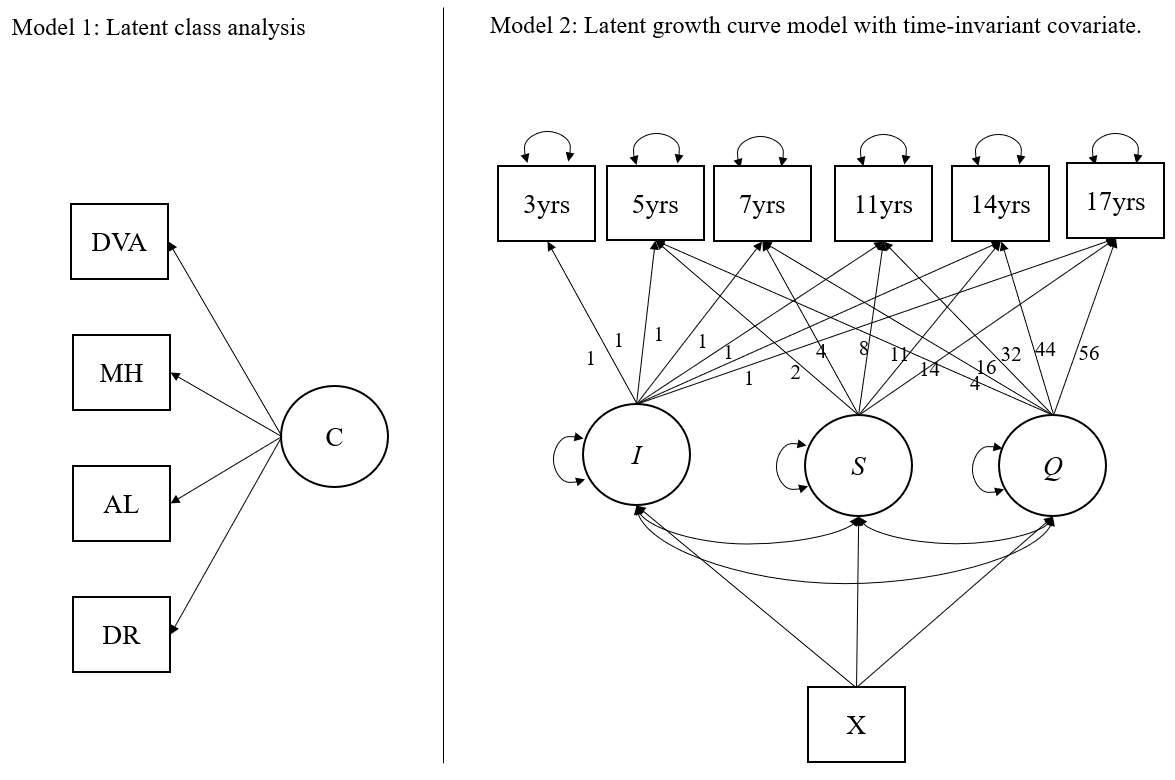
Figure S1.2. Graphical representation of main analyses

Figure S1.1. Graphical representation of the latent variable mixture model. On the left, DVA, MH, AL, and DR represent the four binary outcome variables (manifest indicators) related onto the latent class variable (c). On the right, 3yrs, 5yrs, 7yrs, 11yrs, 14yrs, and 17yrs represent child MH repeatedly measured at six different time periods. *I*, *S*, and *Q* represent the intercept, linear slope, and quadratic slope, respectively. Recursive arrows represent residual errors and X represents child sex. Latent growth curve models with time-invariant covariate were estimated for each latent class.

# Appendix S2. Missing data patterns and comparisons between complete and multiply imputed data

## Table S2.1. Distribution of completeness of household exposure variables at sweep 2 (child three years of age)

|  | **Completeness of each exposure variable for each missing data pattern** | | | | | | | | | | | | | | | |
| --- | --- | --- | --- | --- | --- | --- | --- | --- | --- | --- | --- | --- | --- | --- | --- | --- |
|  | **1** | **2** | **3** | **4** | **5** | **6** | **7** | **8** | **9** | **10** | **11** | **12** | **13** | **14** | **15** | **16** |
| **Parental DVA** | ✓ | 🗶 | 🗶 | 🗶 | ✓ | ✓ | ✓ | 🗶 | 🗶 | 🗶 | 🗶 | ✓ | ✓ | ✓ | ✓ | 🗶 |
| **Parental MH** | ✓ | 🗶 | 🗶 | ✓ | 🗶 | ✓ | 🗶 | ✓ | ✓ | 🗶 | 🗶 | 🗶 | 🗶 | ✓ | ✓ | ✓ |
| **Parental alcohol use** | ✓ | 🗶 | ✓ | ✓ | ✓ | ✓ | 🗶 | ✓ | 🗶 | ✓ | 🗶 | ✓ | 🗶 | 🗶 | 🗶 | 🗶 |
| **Parental drug use** | ✓ | 🗶 | 🗶 | ✓ | 🗶 | 🗶 | 🗶 | 🗶 | 🗶 | ✓ | ✓ | ✓ | ✓ | ✓ | 🗶 | ✓ |
| **Number of participants** | 10,715 | 2,167 | 1,152 | 408 | 357 | 239 | 96 | 52 | 51 | 42 | 41 | 31 | 11 | 8 | 6 | 1 |
| **% Of total sample** | 69.7 | 14.1 | 7.5 | 2.7 | 2.3 | 1.6 | 0.6 | 0.3 | 0.3 | 0.3 | 0.3 | 0.2 | 0.07 | 0.05 | 0.04 | <0.01 |

Key: ✓ =complete exposure variable, 🗶 = incomplete exposure variable.

## Table S2.2. Distribution of completeness of SDQ outcome variables from sweep 2–7 (child 3–17 years of age)

|  | **Completeness of each exposure variable for common missing data patterns** | | | | | | | | | | | | | |
| --- | --- | --- | --- | --- | --- | --- | --- | --- | --- | --- | --- | --- | --- | --- |
|  | **1** | **2** | **3** | **4** | **5** | **6** | **7** | **8** | **9** | **10** | **11** | **12** | **13** | **Other patterns*** |
| **SDQ Total Difficulties Score – Sweep 2** | ✓ | ✓ | ✓ | ✓ | ✓ | ✓ | ✓ | 🗶 | ✓ | ✓ | ✓ | ✓ | ✓ | Various |
| **SDQ Total Difficulties Score – Sweep 3** | ✓ | ✓ | ✓ | 🗶 | ✓ | ✓ | ✓ | ✓ | ✓ | ✓ | 🗶 | ✓ | ✓ | Various |
| **SDQ Total Difficulties Score – Sweep 4** | ✓ | ✓ | ✓ | 🗶 | ✓ | 🗶 | ✓ | ✓ | 🗶 | ✓ | ✓ | 🗶 | ✓ | Various |
| **SDQ Total Difficulties Score – Sweep 5** | ✓ | ✓ | ✓ | 🗶 | 🗶 | 🗶 | ✓ | ✓ | ✓ | 🗶 | ✓ | ✓ | 🗶 | Various |
| **SDQ Total Difficulties Score – Sweep 6** | ✓ | ✓ | 🗶 | 🗶 | 🗶 | 🗶 | 🗶 | ✓ | ✓ | ✓ | ✓ | 🗶 | ✓ | Various |
| **SDQ Total Difficulties Score – Sweep 7** | ✓ | 🗶 | 🗶 | 🗶 | 🗶 | 🗶 | ✓ | ✓ | ✓ | ✓ | ✓ | 🗶 | 🗶 | Various |
| **Number of participants** | 6,305 | 1,691 | 1,076 | 923 | 769 | 769 | 461 | 308 | 308 | 308 | 158 | 158 | 158 | 1,999 |
| **% Of total sample** | 41 | 11 | 7 | 6 | 5 | 5 | 3 | 2 | 2 | 2 | 1 | 1 | 1 | 13 |

Key: ✓ = complete outcome variable, 🗶 = incomplete outcome variable. *In addition to the 13 most common missing data patterns illustrated, there were 51 other missing data patterns all affecting < 1% of total sample (13% of sample in total).

## Table S2.3. Responses rates and percentages for exposure variables and response rates and means and SDs for outcome variables for total sample, complete exposure data sample, incomplete exposure data sample, and imputed sample

| **Exposure and outcome variables** | **Total sample**  **n=15,377** | **Complete^1^**  **n=10,715** | **Incomplete^2^**  **n=4,662** | **Imputed sample^3^**  **n=15,377** |
| --- | --- | --- | --- | --- |
| **Parental DVA** |  |  |  |  |
| No | 10,274 (66.81%) | 9,707 (90.59%) | 567 (12.16%) | 90.2% |
| Yes | 1,189 (7.73%) | 1,008 (9.41%) | 181 (3.88%) | 9.8% |
| Missing | 3,914 (25.45%) | - | 3,914 (83.96%) | - |
| **Parental MH** |  |  |  |  |
| No | 10,844 (70.52%) | 10,242 (95.59%) | 602 (12.91%) | 95.7% |
| Yes | 636 (4.14%) | 473 (4.41%) | 163 (3.50%) | 4.3% |
| Missing | 3,897 (25.34%) | - | 3,897 (83.59%) | - |
| **Parental alcohol use** |  |  |  |  |
| No | 11,214 (72.93%) | 9,244 (86.27%) | 1,970 (42.26%) | 87.2% |
| Yes | 1,782 (11.59%) | 1,471 (13.73%) | 311 (6.67%) | 12.8% |
| Missing | 2,381 (15.48%) | - | 2,381 (51.07%) | - |
| **Parental drug use** |  |  |  |  |
| No | 10,038 (65.28%) | 9,666 (90.21%) | 372 (7.98%) | 90.3% |
| Yes | 1,219 (7.93%) | 1,049 (9.79%) | 170 (3.65%) | 9.7% |
| Missing | 4,120 (26.79%) | - | 4,120 (88.37%) | - |
| **SDQ total difficulties - Sweep 2** | | | | |
| Overall - Mean (SD) | 9.65 (5.33) | 9.39 (5.19) | 10.35 (5.63) | 9.8 |
| Boys - Mean (SD) | 10.16 (5.41) | 9.84 (5.26) | 11.03 (5.73) | 10.3 |
| Girls - Mean (SD) | 9.1 (5.18) | 8.93 (5.08) | 9.61 (5.43) | 9.3 |
| Missing | 1,162 (7.56%) | 275 (2.57%) | 887 (19.03%) | - |
| **SDQ total difficulties - Sweep 3** | | | | |
| Overall - Mean (SD) | 7.3 (4.99) | 7.07 (4.87) | 7.94 (5.25) | 7.5 |
| Boys - Mean (SD) | 7.84 (5.19) | 7.59 (5.08) | 8.49 (5.43) | 8.0 |
| Girls - Mean (SD) | 6.74 (4.71) | 6.53 (4.58) | 7.34 (4.98) | 6.9 |
| Missing | 2,296 (14.93%) | 1,176 (10.98%) | 1,120 (24.02%) | - |
| **SDQ total difficulties - Sweep 4** | | | | |
| Overall - Mean (SD) | 7.45 (5.43) | 7.24 (5.35) | 8.01 (5.61) | 7.6 |
| Boys - Mean (SD) | 8.11 (5.71) | 7.87 (5.64) | 8.77 (5.85) | 8.3 |
| Girls - Mean (SD) | 6.76 (5.04) | 6.59 (4.95) | 7.22 (5.23) | 6.9 |
| Missing | 3,286 (21.37%) | 1,894 (17.68%) | 1,392 (29.86%) | - |
| **SDQ total difficulties - Sweep 5** | | | | |
| Overall - Mean (SD) | 7.67 (5.79) | 7.52 (5.75) | 8.07 (5.89) | 7.8 |
| Boys - Mean (SD) | 8.27 (6.05) | 8.08 (5.97) | 8.79 (6.24) | 8.5 |
| Girls - Mean (SD) | 7.05 (5.45) | 6.95 (5.46) | 7.32 (5.41) | 7.2 |
| Missing | 3,866 (25.14%) | 2,376 (22.17%) | 1,490 (31.96%) | - |
| **SDQ total difficulties - Sweep 6** | | | | |
| Overall - Mean (SD) | 8.1 (5.93) | 7.88 (5.9) | 8.66 (5.97) | 8.3 |
| Boys - Mean (SD) | 8.39 (6.11) | 8.16 (6.08) | 9.0 (6.16) | 8.6 |
| Girls - Mean (SD) | 7.8 (5.73) | 7.6 (5.7) | 8.31 (5.76) | 8.0 |
| Missing | 5,072 (32.98%) | 3,281 (30.62%) | 1,791 (38.42%) | - |
| **SDQ total difficulties - Sweep 7** | | | | |
| Overall - Mean (SD) | 7.44 (5.84) | 7.27 (5.8) | 7.88 (5.93) | 7.8 |
| Boys - Mean (SD) | 7.49 (5.93) | 7.34 (5.92) | 7.9 (5.93) | 7.8 |
| Girls - Mean (SD) | 7.38 (5.76) | 7.2 (5.68) | 7.86 (5.93) | 7.7 |
| Missing | 6,845 (44.51%) | 4,519 (42.17%) | 2,326 (49.89%) | - |

^1^ complete sample includes all families who have complete exposure variables which are used as latent indicators in LCA including household level domestic violence and abuse, mental ill-health, alcohol use, and drug use variables. ^2^ incomplete sample includes all families who have one or more incomplete exposure variables which are used as latent indicators in the LCA including household level domestic violence and abuse, mental ill-health, alcohol use, and drug use variables. ^3^Only percentages are reported for the imputed sample as *n* varies across the 35 imputed datasets.

## Table S2.4. Demographic/contextual characteristics for total sample, complete exposure data sample, incomplete exposure data sample, and imputed sample

| **Demographic/contextual characteristics** | **Total sample**  **n=15,377** | **Complete^1^**  **n=10,715** | **Incomplete^2^**  **n=4,662** | **Imputed sample**  **n=15,377** |
| --- | --- | --- | --- | --- |
| **Child sex** |  |  |  |  |
| Boy | 7,863 (51.13%) | 5,433 (50.7%) | 2,430 (52.12%) | 51.13% |
| Girl | 7,514 (48.87%) | 5,282 (49.3%) | 2,232 (47.88%) | 48.87% |
| Missing | - | - | - | - |
| **Child ethnicity** |  |  |  |  |
| White | 12,708 (82.64%) | 9,617 (89.75%) | 3,091 (66.30%) | 82.64% |
| Mixed | 444 (2.89%) | 303 (2.38%) | 141 (3.02%) | 2.89% |
| Indian | 397 (2.58%) | 171 (1.60%) | 226 (16.58%) | 2.58% |
| Pakistani and Bangladeshi | 1,010 (6.57%) | 237 (2.21%) | 773 (16.58%) | 6.57% |
| Black or Black British | 501 (3.26%) | 256 (2.39%) | 245 (5.26%) | 3.26% |
| Other ethnic group (e.g., Chinese, other) | 218 (1.42%) | 74 (0.69%) | 144 (3.09%) | 1.42% |
| Missing | 99 (0.64%) | 57 (0.53%) | 42 (0.90%) | 0.64% |
| **Siblings in house (including index child)** | | | | |
| One | 3,938 (25.61%) | 2,909 (27.15%) | 1,029 (22.07%) | 25.61% |
| Two | 6,847 (44.53%) | 4,963 (46.32%) | 1,884 (40.41%) | 44.53% |
| Three | 2,943 (19.14%) | 1,937 (18.08%) | 1,006 (21.58%) | 19.14% |
| Four | 1,125 (7.32%) | 653 (6.09%) | 472 (10.12%) | 7.32% |
| Five or more | 524 (3.43%) | 253 (2.36%) | 271 (5.81%) | 3.43% |
| Missing | - | - | - | - |
| **Main respondent age at birth** | | | | |
| <18 years | 382 (2.48%) | 284 (2.65%) | 98 (2.10%) | 2.48% |
| 18-25 years | 4,211 (27.39%) | 2,769 (25.84%) | 1,442 (30.93%) | 27.39% |
| 26-30 years | 4,600 (29.91%) | 3,211 (29.97%) | 1,389 (29.79%) | 29.92% |
| 31-35 years | 4,231 (27.52%) | 3,069 (28.64%) | 1,162 (24.92%) | 27.52% |
| 36-40 years | 1,691 (11%) | 1,203 (11.23%) | 488 (10.47%) | 11.0% |
| >40 years | 261 (1.70%) | 179 (1.67%) | 82 (1.76%) | 1.7% |
| Mean (SD) | 28.68 (6) | 28.8 (6) | 28.41 (6.04) | 28.68 (SE = 0.048) |
| Missing | 1 (0.01%) | - | 1 (0.02%) | - |
| **Partner age at birth*** |  |  |  |  |
| <18 years | 62 (0.49%) | 35 (0.41%) | 27 (0.65%) | 0.5% |
| 18-25 years | 1,790 (14.12%) | 1,039 (12.24%) | 751 (17.94%) | 14.21% |
| 26-30 years | 3,271 (25.80%) | 2,193 (25.83%) | 1,078 (25.75%) | 25.87% |
| 31-35 years | 4,141 (32.67%) | 2,888 (34.01%) | 1,253 (29.93%) | 32.72% |
| 36-40 years | 2,349 (18.53%) | 1,648 (19.41%) | 701 (16.75%) | 18.55% |
| >40 years | 1,031 (8.13%) | 680 (8.01%) | 351 (8.39%) | 8.14% |
| Mean (SD) | 32.02 (6.31) | 32.24 (6.11) | 31.57 (6.68) | 32.01 (SE=0.056) |
| Missing | 33 (0.26%) | 8 (0.09%) | 25 (0.60%) | - |
| **Main respondent ethnicity** |  |  |  |  |
| White | 12,488 (81.21%) | 9,481 (88.48%) | 3,007 (64.50%) | 84.5% |
| Ethnic Minority | 2,135 (13.88%) | 802 (7.46%) | 1,333 (28.59%) | 15.03% |
| Missing | 754 (4.90%) | 432 (4.03%) | 322 (6.91%) | - |
| **Partner respondent ethnicity** | | | | |
| White | 8,757 (69.08%) | 6,802 (80.11%) | 1,955 (46.70%) | 84.64% |
| Ethnic Minority | 1,391 (10.97%) | 510 (6.0%) | 881 (21.05%) | 15.36% |
| Missing | 2,529 (19.95%) | 1,179 (13.89%) | 1,350 (32.25%) | - |
| **Main respondent highest qualification** | | | | |
| Higher degree | 603 (3.92%) | 445 (4.15%) | 158 (3.39%) | 3.93% |
| First degree | 2,055 (13.36%) | 1,587 (14.81%) | 468 (10.04%) | 13.4% |
| Diplomas in higher education | 1,463 (9.51%) | 1,131 (10.56%) | 332 (7.12%) | 9.55% |
| A AS S levels | 1,468 (9.55%) | 1,127 (10.52%) | 341 (7.31%) | 9.58% |
| O level GCSE grades A-C | 5,007 (32.56%) | 3,665 (34.2%) | 1,342 (28.79%) | 32.74% |
| GCSE grades D-G | 1,594 (10.37%) | 1,129 (10.54%) | 465 (9.97%) | 10.48% |
| Other academic qualifications | 447 (2.91%) | 195 (1.82%) | 252 (5.41%) | 2.93% |
| None of these qualifications | 2,662 (17.31%) | 1,400 (13.07%) | 1,262 (27.07%) | 17.44% |
| Missing | 78 (0.51%) | 36 (0.34%) | 42 (0.90%) | - |
| **Partner highest qualification*** | | | | |
| Higher degree | 724 (5.71%) | 553 (6.51%) | 171 (4.09%) | 6.44% |
| First degree | 1,709 (13.48%) | 1,357 (15.98%) | 352 (8.41%) | 14.92% |
| Diplomas in higher education | 1,027 (8.10%) | 808 (9.52%) | 219 (5.23%) | 9.18% |
| A AS S levels | 857 (6.76%) | 653 (7.69%) | 204 (4.87%) | 7.73% |
| O level GCSE grades A-C | 3,476 (27.42%) | 2,634 (31.02%) | 842 (20.11%) | 30.87% |
| GCSE grades D-G | 1,134 (8.95%) | 820 (9.66%) | 314 (7.5%) | 10.57% |
| Other academic qualifications | 378 (2.98%) | 183 (2.16%) | 195 (4.66%) | 3.7% |
| None of these qualifications | 2,234 (17.62%) | 1,238 (14.58%) | 996 (23.79%) | 21.12% |
| Missing | 1,138 (8.98%) | 245 (2.89%) | 893 (21.33%) | - |
| **Family income (banded)** |  |  |  |  |
| £0 - £3,300 | 511 (3.32%) | 348 (3.25%) | 163 (3.50%) | 4.06% |
| £3,300 - £11,000 | 2,546 (16.55%) | 1,825 (17.03%) | 721 (15.47%) | 20.54% |
| £11,000 - £22,000 | 3,831 (24.91%) | 2,628 (24.53%) | 1,203 (25.08%) | 29.74% |
| £22,000 - £33,000 | 2,842 (18.48%) | 2,175 (20.30%) | 667 (14.31%) | 21.54% |
| £33,000 - £55,000 | 2,373 (15.43%) | 1,905 (17.78%) | 468 (10.04%) | 17.77% |
| £55,000+ | 860 (5.59%) | 677 (6.32%) | 183 (3.93%) | 6.34% |
| Missing | 2,414 (15.70%) | 1,157 (10.80%) | 1,257 (26.96%) | - |
| **Poverty (OECD)** |  |  |  |  |
| Above 60% median | 10,167 (66.12%) | 7,534 (70.31%) | 2,633 (56.48%) | 66.92% |
| Below 60% median | 5,029 (32.70%) | 3,163 (29.52%) | 1,866 (40.03%) | 33.08% |
| Missing | 181 (1.18%) | 18 (0.17%) | 163 (3.50%) | - |
| **Housing tenure** |  |  |  |  |
| Own | 9,790 (63.67%) | 7,109 (66.35%) | 2,681 (57.51%) | 64.12% |
| Rent council/housing association | 3,799 (24.71%) | 2,471 (23.06%) | 1,328 (28.49%) | 25.0% |
| Rent private | 1,112 (7.23%) | 772 (7.20%) | 340 (7.29%) | 7.32% |
| Living with parents or other | 539 (3.51%) | 363 (3.39%) | 176 (3.78%) | 3.57% |
| Missing | 137 (0.89%) | - | 137 (2.94%) | - |

*Number of eligible partners in sample; total sample n=12,677, complete sample n=8,491, incomplete sample n=4,186. ^1^ complete sample includes all families who have complete exposure variables which are used as latent indicators in LCA including household level domestic violence, mental ill-health, alcohol use, and drug use variables. ^2^ incomplete sample includes all families who have one or more incomplete exposure variables which are used as latent indicators in the LCA including household level domestic violence, mental ill-health, alcohol use, and drug use variables.

# Appendix S3. Complete exposure data case analyses (n=10,715)

## Table S3.1. Latent class analysis fit indices (complete exposure data)

| **Model fit** | **Number of latent classes estimated** | | |
| --- | --- | --- | --- |
|  | **2 classes** | **3 classes** | **4 classes** |
| **Entropy** | 0.566 | **0.748** | 0.374 |
| **AIC** | 25677.206 | **25653.946** | 25663.034 |
| **BIC** | **25742.721** | 25755.858 | 25801.343 |
| **c-BIC** | 25714.12 | **25711.367** | 25740.964 |
| **VLMR LRT p value** | p < 0.01 | p < 0.01 | p = 0.8342 |
| **BLRT p value** | - | **p < 0.01** | - |

**NB.** AIC = Akaike Information Criterion (AIC); BIC = Bayesian Information Criterion; c-BIC = sample size adjusted BIC; VLMR LRT = Vuong-Lo-Mendell-Rubin Likelihood Ratio Test; BLRT = Bootstrap Likelihood-Ratio Test.

## Table S3.2. Latent classes, item-responses, and membership probabilities for each estimated latent class model (complete exposure data)

| **LCA indicators** | **Latent class item-response probabilities** | | | |
| --- | --- | --- | --- | --- |
|  | **Latent Class 1** | **Latent Class 2** | **Latent Class 3** | **Latent Class 4** |
| **Two-class model** |  |  |  |  |
| **Parental DVA** |  |  |  |  |
| No | 0.627 | 0.955 | - | - |
| Yes | 0.373 | 0.045 | - | - |
| **Parental MH** |  |  |  |  |
| No | 0.884 | 0.968 | - | - |
| Yes | 0.116 | 0.032 | - | - |
| **Parental alcohol use** |  |  |  |  |
| No | 0.740 | 0.884 | - | - |
| Yes | 0.260 | 0.116 | - | - |
| **Parental drug use** |  |  |  |  |
| No | 0.607 | 0.954 | - | - |
| Yes | 0.393 | 0.046 | - | - |
| **Proportions of sample** | 14.86% | 85.14% | - | - |
| **Three-class model (selected model)** | | | | |
| **Parental DVA** |  |  |  |  |
| No | 0.919 | 0.553 | 0.949 | - |
| Yes | 0.081 | 0.447 | 0.051 | - |
| **Parental MH** |  |  |  |  |
| No | 1 | 0.844 | 0.965 | - |
| Yes | 0 | 0.156 | 0.035 | - |
| **Parental alcohol use** |  |  |  |  |
| No | 0 | 0.765 | 0.977 | - |
| Yes | 1 | 0.235 | 0.023 | - |
| **Parental drug use** |  |  |  |  |
| No | 0.903 | 0.565 | 0.945 | - |
| Yes | 0.097 | 0.435 | 0.055 | - |
| **Proportions of sample** | 12.80% | 3.5% | 83.6% | - |
| **Four-class model** |  |  |  |  |
| **Parental DVA** |  |  |  |  |
| No | 0.905 | 0.888 | 0.499 | 0.984 |
| Yes | 0.095 | 0.112 | 0.501 | 0.016 |
| **Parental MH** |  |  |  |  |
| No | 0.931 | 0.994 | 0.839 | 0.994 |
| Yes | 0.069 | 0.006 | 0.161 | 0.006 |
| **Parental alcohol use** |  |  |  |  |
| No | 0.980 | 0.199 | 0.738 | 0.923 |
| Yes | 0.02 | 0.801 | 0.262 | 0.077 |
| **Parental drug use** |  |  |  |  |
| No | 0.908 | 0.874 | 0.502 | 0.974 |
| Yes | 0.092 | 0.126 | 0.498 | 0.026 |
| **Proportions of sample** | 43.52% | 9.98% | 7.0% | 39.5% |

## Table S3.3. Demographic/contextual profiles of each latent class in selected three-class model (complete exposure data)

| **Demographic and contextual variables** | **Conditional probabilities given latent class** | | | **Differences between latent classes^** | | |
| --- | --- | --- | --- | --- | --- | --- |
|  | **Latent Class 1**  ***“High-frequency alcohol use”*** | **Latent Class 2**  ***“Elevated adversity”*** | **Latent Class 3**  ***“Low-level adversity”*** | **Class 1 vs Class 2** | **Class 1 vs Class 3** | **Class 2 vs Class 3** |
| **Child sex** | | | |  |  |  |
| Boy | 52.1% | 52.7% | 50.3% | Chi-squared = 0.03  p-value = 0.867 | Chi-squared = 0.83 p-value = 0.363 | Chi-squared = 0.62 p-value = 0.429 |
| Girl | 47.9% | 47.3% | 49.7% |  |  |  |
| **Number of children in household (including cohort child)*** | | | |  |  |  |
| One | 20.2% | 20.0% | 28.6% | Chi-squared = 5.65  p-value = 0.227 | Chi-squared = 16.65  p-value = 0.002* | Chi-squared = 7.70  p-value = 0.103 |
| Two | 54.2% | 46.9% | 45.4% |  |  |  |
| Three | 18.2% | 21.4% | 17.7% |  |  |  |
| Four | 6.0% | 8.1% | 5.9% |  |  |  |
| Five or more | 1.4% | 3.5% | 2.4% |  |  |  |
| **Main respondent age at birth** | | | |  |  |  |
| <18 years | 0% | 2.4% | 3.0% | Chi-squared = 135.51  p-value = < 0.001* | Chi-squared = 329.68  p-value = < 0.001* | Chi-squared = 57.44 p-value = < 0.001* |
| 18-25 years | 3.1% | 45.3% | 25.8% |  |  |  |
| 26-30 years | 26.2% | 28.9% | 30.6% |  |  |  |
| 31-35 years | 42.8% | 19.4% | 28.3% |  |  |  |
| 36-40 years | 24.4% | 3.7% | 10.7% |  |  |  |
| >40 years | 3.5% | 0.3% | 1.6% |  |  |  |
| **Partner respondent age at birth*** | | | |  |  |  |
| <18 years | 0% | 1.1% | 0.3% | Chi-squared = 228.68  p-value = < 0.001* | Chi-squared = 492.61  p-value = < 0.001* | Chi-squared = 74.67 p-value = < 0.001* |
| 18-25 years | 0% | 26.5% | 10.9% |  |  |  |
| 26-30 years | 12.3% | 34.1% | 26.0% |  |  |  |
| 31-35 years | 38.5% | 23.1% | 35.7% |  |  |  |
| 36-40 years | 31.1% | 12.8% | 19.2% |  |  |  |
| >40 years | 18.0% | 2.4% | 7.8% |  |  |  |
| **Main respondent ethnicity*** | | | |  |  |  |
| White | 98.5% | 94.7% | 91.1% | Chi-squared = 3.60 p-value = 0.058 | Chi-squared = 37.46 p-value = < 0.001* | Chi-squared = 5.49 p-value = 0.019 |
| Ethnic minority | 1.5% | 5.3% | 8.9% |  |  |  |
| **Partner respondent ethnicity*** | | | |  |  |  |
| White | 100.0% | 93.1% | 92.1% | Chi-squared = 19.79  p-value = < 0.001* | Chi-squared = 552.23  p-value = < 0.001* | Chi-squared = 0.35 p-value = 0.555 |
| Ethnic minority | 0.0% | 6.9% | 7.9% |  |  |  |
| **Main respondent highest qualification*** | | | |  |  |  |
| Higher degree | 8.5% | 2.8% | 3.8% | Chi-squared = 43.17  p-value = < 0.001* | Chi-squared = 37.94 p-value = < 0.001* | Chi-squared = 13.21 p-value = 0.067 |
| First degree | 30.2% | 7.9% | 13.8% |  |  |  |
| Diploma | 13.0% | 8.5% | 10.5% |  |  |  |
| A/AS/L Level | 14.1% | 8.8% | 10.3% |  |  |  |
| GCSE A-C | 26.1% | 33.8% | 35.3% |  |  |  |
| GCSE D-G | 3.8% | 13.8% | 11.0% |  |  |  |
| Other | 0.8% | 1.4% | 2.0% |  |  |  |
| None listed | 3.6% | 23.0% | 13.3% |  |  |  |
| **Partner** **respondent highest qualification*** | | | |  |  |  |
| Higher degree | 11.3% | 2.1% | 6.8% | Chi-squared = 24.01  p-value = 0.001* | Chi-squared = 10.22 p-value = 0.177 | Chi-squared = 37.44 p-value = < 0.001* |
| First degree | 26.9% | 7.0% | 16.6% |  |  |  |
| Diploma | 10.3% | 7.8% | 10.0% |  |  |  |
| A/AS/L Level | 10.5% | 7.6% | 7.7% |  |  |  |
| GCSE A-C | 28.8% | 35.5% | 31.8% |  |  |  |
| GCSE D-G | 5.1% | 14.5% | 9.8% |  |  |  |
| Other | 1.2% | 2.6% | 2.3% |  |  |  |
| None listed | 5.9% | 23.0% | 14.9% |  |  |  |
| **Joint annual income*** | | | |  |  |  |
| £0 - £3300 | 1.4% | 4.7% | 3.8% | Chi-squared = 362.39  p-value = < 0.001* | Chi-squared = 1179.65  p-value = < 0.001* | Chi-squared = 52.82 p-value = < 0.001* |
| £3300 - £11000 | 0% | 26.6% | 20.3% |  |  |  |
| £11000 - £22000 | 15.7% | 36.3% | 27.5% |  |  |  |
| £22000 - £33000 | 27.5% | 21.6% | 22.3% |  |  |  |
| £33000 - £55000 | 36.0% | 7.6% | 19.9% |  |  |  |
| £55000+ | 19.4% | 3.3% | 6.1% |  |  |  |
| **Poverty level*** | | | |  |  |  |
| Above 60% poverty level | 99.8% | 51.7% | 69.6% | Chi-squared = 45.79  p-value = < 0.001* | Chi-squared = 34.89 p-value = < 0.001* | Chi-squared = 14.03 p-value = < 0.001* |
| Below 60% poverty level | 0.2% | 48.3% | 30.4% |  |  |  |
| **Housing tenure** | | | |  |  |  |
| Own | 96.7% | 34.8% | 67.7% | Chi-squared = 185.61  p-value = < 0.001* | Chi-squared = 383.45  p-value = < 0.001* | Chi-squared = 46.49 p-value = < 0.001* |
| Rent council / housing association | 0% | 46.6% | 22.1% |  |  |  |
| Rent private | 2.8% | 15.6% | 6.4% |  |  |  |
| Living with parents and other | 0.6% | 3.0% | 3.7% |  |  |  |
| **NB.** Complete case analysis based on LCA including 10,715 participants. Partner respondent age n=8,483; main respondent ethnicity n=10,283; partner respondent ethnicity n=7,312; main respondent highest qualification n=10,679; partner respondent highest qualification n=8,246; income n=9,558; poverty n=10,697. ^Significance was determined based on an adjusted alpha value of 0.017 (i.e., 0.05/3) to account for multiple testing. | | | | | | |

## Table S3.4. Unconditional, conditional, and latent-class-specific latent growth curve models (complete exposure data)

|  | **Overall latent growth curve model** | | | | | | | | **Latent-class-specific models** | | | | | |
| --- | --- | --- | --- | --- | --- | --- | --- | --- | --- | --- | --- | --- | --- | --- |
|  | **Model 1** | | **Model 2** | | **Model 3** | | **Model 4** | | **Latent class 1 *“High-frequency alcohol use”*** | | **Latent class 2**  ***“Elevated adversity”*** | | **Latent class 3**  ***“Low-level adversity”*** | |
|  | **Estimate** | **SE** | **Estimate** | **SE** | **Estimate** | **SE** | **Estimate** | **SE** | **Estimate** | **SE** | **Estimate** | **SE** | **Estimate** | **SE** |
| **Mean** |  |  |  |  |  |  |  |  |  |  |  |  |  |  |
| Intercept | 1.972* | 0.006 | 2.063* | 0.006 | 2.140* | 0.006 | 2.192* | 0.008 | 2.050* | 0.023 | 2.261* | 0.014 | 2.200* | 0.009 |
| Linear slope | - | - | -0.015* | 0.001 | -0.066* | 0.002 | -0.055* | 0.002 | -0.055* | 0.007 | -0.045* | 0.004 | -0.056* | 0.003 |
| Quadratic slope | - | - | - | - | 0.004* | <0.001 | 0.003* | <0.001 | 0.003* | 0.001 | 0.002* | <0.001 | 0.003* | <0.001 |
| **Variance** |  |  |  |  |  |  |  |  |  |  |  |  |  |  |
| Intercept | 0.274* | 0.005 | 0.238* | 0.005 | 0.207* | 0.005 | 0.205* | 0.005 | 0.178* | 0.014 | 0.207* | 0.009 | 0.205* | 0.006 |
| Linear slope | - | - | 0.001* | <0.001 | 0.008* | <0.001 | 0.008* | <0.001 | 0.006* | 0.002 | 0.007* | 0.001 | 0.008* | 0.001 |
| Quadratic slope | - | - | - | - | <0.001* | <0.001 | <0.001* | <0.001 | <0.001* | <0.001 | <0.001* | 0.001 | <0.001* | <0.001 |
| **Covariance matrix** | | | | | | | | | | | | | | |
| Intercept with linear | - | - | -0.001† | <0.001 | 0.008* | 0.001 | 0.008* | 0.001 | 0.014* | 0.004 | 0.006† | 0.002 | 0.007* | 0.001 |
| Intercept with quadratic | - | - | - | - | -0.001* | <0.001 | -0.001* | <0.001 | -0.001* | <0.001 | -0.001* | <0.001 | -0.001* | <0.001 |
| Linear with quadratic | - | - | - | - | 0.000* | <0.001 | <0.001* | <0.001 | <0.001* | <0.001 | <0.001* | <0.001 | <0.001* | <0.001 |
| **Child sex (girls)** | | | | | | | | | | | | | | |
| Intercept | - | - | - | - | - | - | -0.105* | 0.011 | -0.095† | 0.031 | -0.102* | 0.021 | -0.106* | 0.012 |
| Linear slope | - | - | - | - | - | - | -0.021* | 0.003 | -0.025† | 0.010 | -0.024* | 0.006 | -0.021* | 0.004 |
| Quadratic slope | - | - | - | - | - | - | 0.002* | 0.002 | 0.002* | 0.001 | 0.002* | <0.001 | 0.002* | <0.001 |
| **Fit indices** |  |  |  |  |  |  |  |  |  |  |  |  |  |  |
| χ2 (df) | 6539.568 (19)* | | 4160.850 (16)* | | **2134.847** (12)* | | 2140.161 (15) | | - | - | - | - | - | - |
| RMSEA (CI) | 0.179 (0.176 – 0.183) | | 0.156 (0.152 – 0.160) | | 0.129 (0.124 – 0.133) | | **0.115** (0.111 – 0.119) | | - | - | - | - | - | - |
| CFI | 0.727 | | 0.826 | | 0.911 | | **0.912** | | - | - | - | - | - | - |
| SRMR | 0.275 | | 0.171 | | 0.076 | | **0.068** | | - | - | - | - | - | - |

* p≤0.001 † p<0.05. Model 1 = Unconditional model, random intercept only (fixed slope); Model 2 = Unconditional model, random intercept and linear slope; Model 3 = Unconditional model, random intercept, linear, and quadratic slope; Model 4 = Conditional model (including child sex as covariate), random intercept, linear, and quadratic slope.

## Table S3.5. Wald test of parameter constraints for overall, intercept, and linear and quadratic slope (complete exposure data)

|  | **Overall** | | | **Intercept** | | | **Linear and quadratic** | | |
| --- | --- | --- | --- | --- | --- | --- | --- | --- | --- |
|  | **Estimate** | **df** | **p** | **Estimate** | **df** | **p** | **Estimate** | **df** | **p** |
| **Omnibus** | 116.116 | 6 | <0.001 | 77.214 | 2 | <0.001 | 8.807 | 4 | 0.0661 |
| **Latent class 1 vs latent class 2** | - | - | - | 74.381 | 1 | <0.001 | - | - | - |
| **Latent class 1 vs latent class 3** | - | - | - | 38.595 | 1 | <0.001 | - | - | - |
| **Latent class 2 vs latent class 3** | - | - | - | 18.721 | 1 | <0.001 | - | - | - |

# Appendix S4. Multiply imputed data analyses (n=15,377)

## Table S4.1. Covariance matrix for unconditional and latent-class-specific latent growth curve models (multiply imputed data)

|  | **Overall latent growth curve models** | | | | | | | | **Latent-class-specific models** | | | | | |
| --- | --- | --- | --- | --- | --- | --- | --- | --- | --- | --- | --- | --- | --- | --- |
|  | **Model 1** | | **Model 2** | | **Model 3** | | **Model 4** | | **Latent class 1 *“High-frequency alcohol use”*** | | **Latent class 2**  ***“Elevated adversity”*** | | **Latent class 3**  ***“Low-level adversity”*** | |
|  | **Estimate** | **SE** | **Estimate** | **SE** | **Estimate** | **SE** | **Estimate** | **SE** | **Estimate** | **SE** | **Estimate** | **SE** | **Estimate** | **SE** |
| **Covariance matrix** | | | | | | | | | | | | | | |
| Intercept with linear | - | - | -0.003* | <0.001 | 0.007* | 0.001 | 0.006* | 0.001 | 0.012* | 0.003 | 0.006* | 0.002 | 0.006* | 0.001 |
| Intercept with quadratic | - | - | - | - | -0.001* | <0.001 | -0.001* | <0.001 | -0.001* | <0.001 | -0.001* | <0.001 | -0.001* | <0.001 |
| Linear with quadratic | - | - | - | - | <0.001* | <0.001 | <0.001* | <0.001 | <0.001* | <0.001 | <0.001* | <0.001 | <0.001* | <0.001 |
| * p≤0.001 † p<0.05. Model 1 = Unconditional model, random intercept only (fixed slope); Model 2 = Unconditional model, random intercept and linear slope; Model 3 = Unconditional model, random intercept, linear, and quadratic slope; Model 4 = Conditional model (including child sex as covariate), random intercept, linear, and quadratic slope, obtained as main coefficients | | | | | | | | | | | | | | |

## Table S4.2. Wald test of parameter constraints for overall, intercept, and linear and quadratic slope (multiply imputed data)

|  | **Overall** | | | **Intercept** | | | **Linear and quadratic** | | |
| --- | --- | --- | --- | --- | --- | --- | --- | --- | --- |
|  | **Estimate** | **df** | **p** | **Estimate** | **df** | **p** | **Estimate** | **df** | **p** |
| **Omnibus** | 118.598 | 6 | <0.001 | 90.126 | 2 | <0.001 | 4.606 | 4 | 0.3302 |
| **Latent class 1 vs latent class 2** | - | - | - | 90.08 | 1 | <0.001 | - | - | - |
| **Latent class 1 vs latent class 3** | - | - | - | 55.4 | 1 | <0.001 | - | - | - |
| **Latent class 2 vs latent class 3** | - | - | - | 11.381 | 1 | <0.001 | - | - | - |

# References

1. Galbally M, Lewis AJ. Depression and parenting: the need for improved intervention models. Curr Opin Psychol. 2017;15:61-5.

2. Adjei NK, Schlüter DK, Straatmann VS, Melis G, Fleming K, McGovern R, et al. Impact of poverty and family adversity on adolescent health: a multi-trajectory analysis using the UK Millennium Cohort Study. The Lancet Regional Health - Europe. 2021;13:100279.

3. Straatmann VS, Lai E, Law C, Whitehead M, Strandberg-Larsen K, Taylor-Robinson D. How do early-life adverse childhood experiences mediate the relationship between childhood socioeconomic conditions and adolescent health outcomes in the UK? Journal of Epidemiology and Community Health. 2020;74(11):969.

4. Bevilacqua L, Kelly Y, Heilmann A, Priest N, Lacey RE. Adverse childhood experiences and trajectories of internalizing, externalizing, and prosocial behaviors from childhood to adolescence. Child Abuse & Neglect. 2021;112:104890.

5. Goodman R. Psychometric properties of the strengths and difficulties questionnaire. J Am Acad Child Adolesc Psychiatry. 2001;40(11):1337-45.

6. Goodman R, Ford T, Simmons H, Gatward R, Meltzer H. Using the Strengths and Difficulties Questionnaire (SDQ) to screen for child psychiatric disorders in a community sample. Int Rev Psychiatry. 2003;15(1-2):166-72.
